# The African Transport Systems Database - a geospatial database of multi-modal connected networks

**DOI:** 10.1038/s41597-025-06483-7

**Published:** 2025-12-24

**Authors:** Silvia Colombo, Raghav Pant, Marcus Young, Fred Thomas, Tom Russell, Jasper Verschuur, Jim W. Hall

**Affiliations:** 1 Environmental Change Institute, University of Oxford, Oxford, UK; 2 Transportation Research Group, University of Southampton, Southampton, UK; 3 Faculty of Technology, Policy and Management, Delft University Technology, Delft, Netherlands

**Keywords:** Climate-change impacts, Developing world, Geography, Sustainability

## Abstract

We present the first comprehensive geolocated multi-modal transport database for the whole continent of Africa, the African Transport Systems Database (AfTS-Db), including road, rail, aviation, maritime and inland waterway networks. To do so, we created and standardized asset and network data across all transport modes, including inter-modal connections, attributes of road and rail corridors and estimated annual statistics for airports and ports. The African Transport Systems Database includes 234 airports including their airline routes, 179 maritime ports and their connections with each other, 132 inland ports and docking sites with river and lake connections, 4,412 railway stations connected across 99,373 kilometers of rail lines, and 1,004,512 kilometers of roads mainly comprised of all motorways, trunk roads, primary and secondary routes across Africa and some local roads that connect to other transport modes. The AfTS-Db provides key information for transport planning, resilience assessments, asset management and development of transport models and applications. Furthermore, we expect the data will also be of relevance for environmental, health, social and economic studies.

## Background

Transport networks are lifeline systems that enable mobility of people and facilitate the movement of goods, thereby enabling development benefits and economic growth^1,2^. The expansion and modernization of these transport networks form part of most countries’ economic development strategies, especially for countries in Africa^3^. Transport infrastructure investment, by public bodies and private investors, in Africa has been steadily increasing in monetary value over the past decades^4^. For example, investments in port projects across Africa with private participation grew between 2012–2022 to US$ 12.7 billion from US$ 7.3 billion between 2002–2012^5^. This aligns with the African Continental Free Trade Area (AfCFTA) project agenda for 2063 to create a single market of 1.3 billion people and a combined GDP of US$ 3.4 trillion across 55 African countries. Investments in transport infrastructure are critical to achieve this, particularly to support industrialization, job creation, and enhanced competitiveness^6,7^. Recognizing this need, the African Development Bank (AfDB) has invested over US$ 13 billion between 2004 and 2022 in regional strategic road corridor projects to improve connectivity, strengthen market accessibility across countries and integrate supply chains^8^. This is complemented by other investment initiatives such as, amongst others, the World Bank (approximately US$ 2 billion approved investments in transport projects between 2006 and 2024)^9–14^, The Global Gateway Africa – Europe Investment Package (which aims to invest € 150 billion between 2021–2027 in various initiatives including strategic transport corridor development in Africa)^15^, China’s Belt and Road initiative (which has invested US$ 53 billion in transport projects across Africa between 2000–2023)^16,17^ and United States’ (US) investments in the Lobito corridor in Southern Africa (US$ 1 billion)^18^.

Though the initiatives mentioned above are bringing new investments in Africa’s transport sector, the existing investment is well below Africa’s transport needs which have been unable to keep up with the continent’s increasing population, urbanization and trade^19^. Estimates suggest that the transport investment needs in Africa in 2020 were between US$ 37–49 billion compared to the actual commitment of US$ 33 billion, resulting in an investment gap of US$ 4–16 billion^20^. Transport investments in Africa have mostly concentrated around maritime ports to enhance existing or new hubs, while the integration with the road and rail linkages has been underfunded and underdeveloped^19^.

While improvement and development of transport networks can lead to economic growth, regional integration and market expansion, the design and planning of such infrastructure needs to be integrated with considerations of their resilience to climate change, biodiversity and environmental impacts, CO_2_ emissions, social exclusion and displacement^21,22^. Transport infrastructures development and quality improvements have been shown to have positive impacts on intra-Africa trade and movements of people^23^. To some extent wider social benefits of integrated transport corridor development in Africa is also seen, enabling access to schools, health facilities, communities, supporting local businesses and households, and consequently supporting the general social welfare^24^. However, there is also compelling evidence to suggest that ongoing large-scale transport corridor developments are causing social exclusion where these corridors do not arrive for marginalized groups or do not benefit vulnerable groups^25^. Research also suggests that there is low integration of future climate risks and adaptation planning across transport projects in East Africa^26^, which might lock-in patterns of development in undesirable ways leading to maladaptation^27^.

To make the case for improving transport investments in Africa and to adequately assess, and mitigate, their impacts, there is a need for open-access data that maps the existing and planned transport networks together^26,28^. Such data needs to be geospatial, on an asset level, and establish connectivity between assets, in order to provide a comparison of the patterns of development across Africa. Elsewhere, some global and continent level initiatives have adopted geospatial network approaches, such as the Global Infrastructure Impact Viewer^29^, the European Transport Maps^30^ and TENtec Information System^31^, the Asian Transport Observatory^32^, to inform the assessment and management of strategic road and rail corridors^33^. Providing integrated spatial network data could help demonstrate how new developments would affect trade and passenger mobility choices in the future and link economic centers and investment areas to regional markets and ports. However, for Africa there is a general lack of Geographical Information Systems (GIS) data that is standardized and supported by a modelling process that utilizes reliable open-access information and could be updated in the future^34^.

## Existing databases and platforms for Africa

Various platforms and research papers provide valuable data on Africa’s transport sector. However, they do not encompass the entire continent or lack information necessary for geospatial mapping of existing assets and new developments. A brief description of these studies and their content and limitations is provided below and listed in Supplementary Table S1.

The UNCTADstat database managed by the United Nations Conference on Trade and Development (UNCTAD) provides trade and transport statistics for African countries, including data on multimodal freight movements^35^. Mphigalale (2020)^36^ conducted both qualitative and quantitative analyses to evaluate private and public infrastructure investment needs and challenges in three countries – Angola, Democratic Republic of Congo and Ghana. However, these datasets are not spatial, limiting their use for planning purposes. At the national scale, some literature is found that assesses railway information, in Ghana^37^ and Nigeria^38,39^. In Ethiopia, Аmah (2023)^40^ characterized the existing state of the main types of land transport (road and rail), and analyzed external and internal factors influencing the formation of variants of the multimodal transport network scheme. These studies either consider old versions of network datasets or are limited to national scales only.

The African Regional Integration Index (ARII) seeks to assess the status of electricity, transportation, information and communication technologies, and water and sanitation at the national and regional level^41^. The data is currently available only at the national level and lacks a detailed geographical breakdown^42^. Similarly, the African Infrastructure Development Index (AIDI) tracks progress of infrastructure development across countries, through high-level non-spatial statistics^43^. The African Infrastructure Knowledge Portal^44^ provides limited access to rail data and open access to ports and air transport data by facility at the continental level, reporting the capacity (people or tons of material) and transported freight value. The OurAirport^45^ project is an open-source, global airports geodatabase with size specifications (very small, small, medium, large and other smaller airports). It is continuously updated by users, and it is a very complete dataset in terms of number of airports, while it does not contain additional information on internal and international routes or some quantified measure of usage (e.g. flight numbers or passenger seats offered on flights).

A number of AfDB supported initiatives provide map-based information on the ongoing transport development projects in Africa. MapAfrica maps the various transport projects and associates them with temporal and technical data on the risk category, funders, and beneficiaries of each project^46^. However, it omits the actual spatial coverage of projects, often giving only a point location of the region where the project is happening. The African Infrastructure Database (AID) spatially tracks transport projects, but it does not provide a sense of how these projects integrate with the rest of transport networks in Africa^47,48^. Important cross-border road corridors in the African continent are identified by two African Development Bank reports, complete with characteristics such as length and costs of the projects, together with maps illustrating their development in space. This information is useful for understanding continental integration of road corridors, though it is not available as a GIS data resource^8,49^. Thorn *et al*.^28^ (https://datadryad.org/dataset/doi:10.5061/dryad.9kd51c5hw)^28^ first mapped development corridors in Africa, creating a comprehensive open-source geo-database of 184 projects, including railways, ports, pipelines, airports, techno-cities, and industrial parks. The database, with 22 attributes, synthesizes data from multiple sources and has also served as a valuable resource for our work. Similarly, the AidData initiative has provided a useful global open-source spatial dataset mapping and tracking China’s development Aid projects linked with OpenStreetMap information^50^. However, these studies and datasets also do not integrate the projects with the existing networks in Africa. A database compiled by the United States Geological Survey (USGS), focusing on the mineral industries and related infrastructure in Africa^51^, includes detailed layers of ports, roads, and railways across the continent. However, the USGS database also does not provide much information on the road and rail network attributes and their connectivity to mines and ports, which are provided in our database.

## Aim of the current work

Given that the available data on transportation infrastructure is both sparse and fragmented, a comprehensive open dataset is urgently needed for planning future projects and conducting high level studies supporting more analysis across the African continent. This lack of comprehensive data presents significant challenges for researchers, policymakers, and developers who are trying to understand the broader transportation landscape. Without detailed information on existing infrastructures - such as their condition, usage statistics, and connectivity - it becomes difficult to make informed decisions about where investments are most needed. Moreover, this fragmented approach undermines the potential for regional collaboration and integration of transport networks, which could greatly enhance efficiency and economic growth. To foster development and improve transportation across Africa, a more unified and detailed dataset is essential. Such data would not only facilitate better planning and resource allocation but also support the creation of social, environmentally and economically sustainable transport solutions that meet the diverse needs of the continent’s growing population.

To address the above needs, this paper presents a novel database of multimodal transport networks (roads, railways, aviation, inland and maritime waterways) for Africa, combining known open spatial datasets with additional information extracted from reports, including existing and approved and already financed infrastructure projects (see Supplementary Table S2). These transport networks are conceptualized as the collection and interconnection of physical assets that operate in a coordinated way to provide specific infrastructure services^27^. The networks consist of nodes (representing key point locations of physical facilities such as road junctions, railway stations, ports and airports) and links (representing physical connections between nodes such as road sections, railway lines, airline and waterway routes). The multimodal transport system is then created by identifying nodes based on their functionality and proximity to create a network-of-networks^52,53^.

## Methods

The Africa Transport Systems Database (AfTS-Db) is a geolocated interconnected transport asset and network database for the African continent. The steps to create the AfTS-Db are as follows:

1. Create workflows to gather existing open-access reliable transport GIS data for the entire African continent for different transport modes (roads, railways, waterways and airlines).
2. Collect attributes of interest, to identify names, physical characteristics and usage information of important assets across transport modes.
3. Create and assemble spatial information on major road corridors and rail routes, together with airports and ports, from various African countries.
4. Standardize and integrate all datasets to a common network specification, specifying nodes and edges of each transport mode.
5. Create multimodal connections across the different transport networks.

## Search strategy and data manipulation

**Fig. 1:**
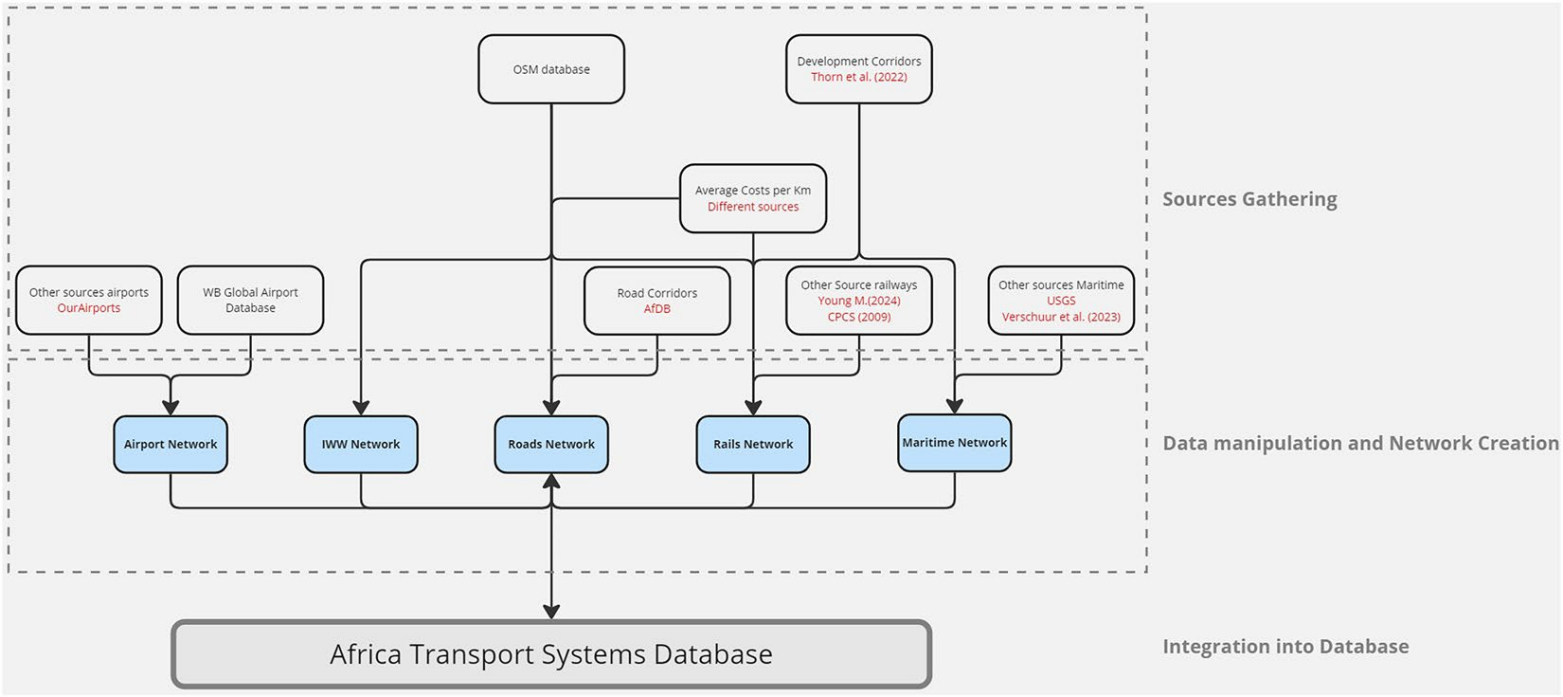
Outline of the structure followed to build the multimodal datasets, starting from different input datasets, followed by individual cleaning and network creation of each transport mode, and finally integrating them together with standardized column names.

The development of the AfTS-Db follows a structured approach to ensure accuracy, consistency, and comprehensiveness (see Fig. 1). For roads and railways networks, the process begins with extraction of data from OpenStreetMap (OSM)^54^, converting it into topological networks and enriching attributes in the OSM data with other relevant reports and datasets where geographic and network-related data are gathered. OSM is an open-source, open-contributors dataset mapping the world, particularly useful for road and rail infrastructures geographical data and widely used for the transport sector analysis^55^. New or additional projects from other sources^28,47,50^ are added to the OSM networks. Further attributes, such as names of specific road corridors and railway lines, railway station facilities based on the type of usage (e.g. a dry port station or a station at a mine location) are added to OSM network assets (nodes and edges) from different sources^8,49,56^. For waterways and airlines networks other known open datasets (see below) are gathered and different attributes from these datasets are combined to create consistent datasets. Each transport mode dataset consists of a node layer (for point assets) and an edge layer (for line connections) with common column names and attributes that are standardized across all modes (see Table 2).

This sector-wise network data creation is followed by the creation of multimodal connections, to represent the links between the different nodes of different transport modes. Multimodal connections are inferred based on spatial proximity of the ports and airports to roads and specific railway nodes (based on usage type of the railway station – see Section on multimodal connections below) and by also linking specific stations (see Section on multimodal connections below) to nearest road nodes. To maintain uniformity across datasets, standardization of column names for similar types of attributes are applied, aligning different data sources into a cohesive structure. Additionally, for road and rail assets, estimates of construction and rehabilitation costs from different studies are compiled and incorporated into the dataset. This process provides a high-level assessment of investment costs for existing and planned road and railway projects (see Generalized construction section).

## Airport Network

The World Bank Global Airports database (https://datacatalog.worldbank.org/search/dataset/0038117/Global-Airports)^57^ is used to extract airports across the African continent. The database contains the main airport locations mapped from an air traffic flow repository, which identifies international airports and their flight connections with all other airports globally. Each airport is also attributed with total annual seats offered across all flights that land and depart that airport, which can be considered to be a proxy for annual passenger volumes at the airport. The annual seat numbers are reported for the most recent reported year, which in the dataset is 2019. The total seats between airport pairs are also estimated in the original dataset, providing an understanding of the air traffic flow volumes between airports. However, location accuracy (xy-coordinates) of airports in this dataset is not very good, with some airports found to be several kilometers away from their actual locations when compared with satellite imagery. To correct the locations OurAirports database (https://ourairports.com/data/)^45^ (which has higher positional accuracy for the runways) is cross checked against the World Bank database. Further corrections to the airport locations are made by manually checking with satellite imagery to make sure that the airports’ point locations are at the main terminals. The network topology (airport (nodes) and their connecting flight route (edges)) information is already in the database, the data cleaning process focuses on standardizing column names and order, matching country names with their respective continents and filtering for African airports and creating routes (as straight-line segments between airport pairs with known flight routes) within the continent. It is noted that the OurAirports database^45^ includes several smaller airports spread across Africa, which are ignored. The coverage of main airports with consistent data on the reported routes and total number of seats for each airport is considered more appropriate for the scope of the current database. The original OurAirports^45^ point locations are also maintained as a separate airport layer within the database, where the International Air Transport Association (IATA)^58^ code column serves as the key for matching each airport terminal to its counterpart in the World Bank dataset.

## Maritime Network

The maritime infrastructure network (with port and maritime (i.e. offshore) nodes and edges) is extracted from Verschuur *et al*.^59^ and PortWatch (https://portwatch.imf.org/datasets/acc668d199d1472abaaf2467133d4ca4/about)^60^ (extracted in June 2025), supplemented with some new African continental-level port information^51^ and ports included in the planned development corridors reported by Thorn *et al*.^28^. Some of the nodes and edges along the Suez Canal are incorporated from OSM to represent the physical routes.

The combined Africa specific network resulting from merging the above datasets is created to be consistent with the Verschuur *et al*.^59^ global database, by assigning ID numbers to the additional new ports (not in the global network) that follow the existing numbering convention in the global database. Also, the new ports are linked to the global maritime network by adding new edges in the existing maritime network file. To ensure data integrity, a cleaning process is performed to remove duplicate values, correct geometries, and add country codes as per ISO3166 standard^61^. Finally, an Africa port-to-port routable network is extracted from the global network, by finding the shortest distance navigable routes between all ports in Africa.

Further usage attributes are also added to the port nodes, from the study of Verschuur *et al*.^59^ and PortWatch^60^. The attributes, derived from observed maritime Automatic Identification System (AIS) data on cargo vessel properties and locations tracked from 2019–2024, correspond to information for five types of cargo shipments arriving and departing at each port – container, dry bulk, tankers, Roll-on-Roll-off (RoRo) and general. These include estimates of: (1) annual average number of vessels of different cargo types handled at each port; (2) annual average total weights (in tons) of different cargo type vessels arriving at each port; (3) the average time spent by each cargo type at each port; (4) the total proportion of a country’s exports and imports handled at each port.

## Inland Waterways (IWW) Network

Inland waterways and ports are sparsely used in Africa, with the main components of navigable waterways and ports being concentrated on the Lakes (Victoria, Tanganyika and Malawi) and along waterways and lake of the Congo and Nile river systems^62^. Information on known ports and other docking points (piers, docks, landing sites, and yacht clubs for recreation) along the Nile river are identified from a study on the Nile Basin Initiative (NBI) that explores the feasibility of enhancing the inland waterway transport for goods from the source of the Nile in Lake Victoria to the Mediterranean ports in Egypt^63^. The locations of these ports and docking points are checked and corrected by validating them against satellite data. Other known ports along Lake Victoria, Tanganyika, Malawi and the Congo river are compiled from previous studies^64,65^ and geolocated manually with satellite data. Data on navigable courses along the Congo and Nile rivers is collected from an OSM extract from August 2025 (https://africageoportal.maps.arcgis.com/home/item.html?id=82232d0415c04e7086414dff7eb1310f)^66^, along with known routes information between lake ports^64,67^. The ports and docking points are connected via straight-line edges between connecting lake ports, while ports and docking points along the rivers are connected by routing along the OSM rivers. Unfortunately, there is no credible information on port usage across whole of Africa, so our dataset only contains the node and edge information with network topology. Data are standardized by ensuring uniformity in port names and ISO3166 country codes.

## Rail Network

A topologically connected rail network for the African continent is developed, starting from an OpenStreetMap (OSM) extract from November 2021^68^. The initial extract and data processing uses the Open-GIRA open-source Python-based workflow^69^. This workflow extracts rail data for the whole of Africa from the OSM planet file. The original OSM extract contained disjointed layers of line geometries for rail line and point geometries for locations (e.g. stations, junctions). The workflow then uses the spatial network library snkit^70^ to snap points to lines and establish connectivity between locations to create topological networks. It is noted that the Open-GIRA workflow is automated to download an OSM extract at any time and convert it into a topological network. Though the extract used for this database was a bit dated, it has been updated to reflect recent developments as explained next.

Further refinement is made to the OSM rail network by conducting a thorough review of rail networks across Africa, thereby enriching the OSM network with additional attributes. For the nodes layer useful additional attributes include: (1) names of important stations and stops not found in OSM; (2) a facility tag to identify the type of facility (container terminal, dry port, mine, etc.) being served by the rail node (see Supplementary Fig. S3). For the edges layer important attributes includes: (1) names of all rail lines; (2) status of lines (active, planned, proposed etc.); (3) gauge width for all lines; and (4) lengths of rail segments. Several new lines are also added to the existing OSM network based on reviews of country specific development plans. This process is documented in detail here: https://github.com/trg-rail/africa_rail_network^56^. In addition to this work, final refinement of the rail network is done by adding some more new projects that were found to be spatially mapped in other data sources, such as the Africa corridor database from Thorn *et al*.^28^ and the African Union – Programme for Infrastructure Development in Africa (AU-PIDA) portal (https://www.au-pida.org/pida-projects/)^47^, together with few more planned and proposed rail projects, which seemed to be likely to be developed in the future^71^.

## Road Network

A topologically connected road network for the African continent is developed using an extract of OpenStreetMap (OSM) data from August 2025. This also uses the Open-GIRA open-source workflow^69^, downloading and extracting data on road sections from OSM and adding topology to create routable and connected road networks. Road nodes in this case are created at the junctions and end points of road segments. The workflow provides similar functionality to the widely used OSMnx Python package^72^ which can also extract and create topologically connected road networks from OSM.

From the whole road network for Africa, the main roads are filtered and selected based on their highway classifications (*trunk*, *motorway*, *primary*, and *secondary*), and a small set of minor classification roads are integrated into the network to maintain connections with important locations in other modes of transport - airports, ports, and railway stations. While the Open-GIRA workflow can create a topological network including every level of OSM highway class, the rationale for selecting this filtered version of the road network is to show roads (trunk, motorway and primary) that contribute to significant national corridors, roads (secondary) that contribute to sub-national connectivity and roads that connect to other modes of transport.

For the edges layer, other attributes are extracted from OSM such as the highway classification, surface type, number of lanes, asset type (bridge or no bridge) and speed. While OSM provides some values on surface type and number of lanes for roads, most roads generally have no values. Gap filling is done to infer the road surface type for blank values to create a new column (material) by assuming that the motorways, trunk and primary roads would have an asphalt road surface while other classes of road would have gravel surfaces. Another column for number of lanes is created to sense check and clean given lane numbers in OSM and gap fill remaining values by assuming that motorways, trunk and primary roads would have two lanes while other classes of roads would have a single lane. Some OSM values for the number of lanes are identified as outliers, and a maximum of eight lanes is assumed for such cases. If a segment has more than eight lanes, its lane count is adjusted to match those of the preceding road segment. A column for calculated road segment length is added, leading to the final creation of the road network. In the nodes layer the country code of the location is also added as per ISO3166 codes, which are then transferred to the topology information in the edges layer to infer if a road segment is contained with a single country or traversed two countries – i.e. a border crossing.

OSM extracted data do not provide any information on the name of specific corridors, which are important to have from a development perspective, considering the amount of investment being made in Africa on road corridors (as was discussed in the Background section). Corridor information from the African Development Bank^8,41^ reports is extracted and complemented with some information from the Tripartite Transport and Transit Facilitation Programme (TTTFP)(https://tttfp.org/corridors/all-corridors/)^73^, geographically digitized, and incorporated into the database. This process involves identifying the latitudes and longitudes of the points forming these corridors and determining the roads that connect them, which generally are the main (trunk, motorway, primary) roads in OSM. Where main roads of the OSM dataset do not directly connect with identified corridor locations, minor classification roads are added under the assumption that they will have to be upgraded and included in the corridors in the near future.

## Multimodal Connections

Following the creation of the various transport networks, multimodal connections are inferred between specific point locations (nodes) of different modes. This process creates a dataset of segments (edges) that connects nodes across networks. It is noted that a multimodal edge represents a notional linkage between two nodes and not an actual physical asset. For example, an edge connecting a port node to its nearest road junction node would be called a multimodal edge as it would be the most likely transfer point to move people and goods from the port onto the road network, and vice versa.

Some generalized criteria are applied to create multimodal edges. Ports (maritime and inland) and airports are connected to their nearest road junctions (nodes), based on the assumptions that such connectivity would lead to the nearest accessible roads from ports and airports. Specific railway station facilities serving ports and airports are identified and a multimodal connection is established between the two (see Supplementary Fig. S3).

Connectivity between rail and road networks is established specifically for the purposes of distinguishing freight transfer locations from more general freight and/or passenger transport (see Supplementary Fig. S3 for specific freight transport locations). Specific railway stations for freight are identified based on the following facility tags attached to them – *agriculture, coal terminal*, *container terminal, food production, food storage, freight terminal, freight marshalling yard, fuel terminal, industrial area, manufacturing, military, port, port (dry), port (river), port (inland), road-rail transfer, storage*, and assigned to a *freight* usage type (usage_type column in the dataset). These types of station facilities signify real locations where freight would be moved between rail and road networks. The remaining categories of facility are assigned to as passenger usage type, to facilitate movement of people along the rail network. Airports, inland and maritime ports handle both freight and passengers, so their connections to other modes are tagged as a more general *freight/passenger* usage type.

The resulting multimodal database contains only edge elements, each assigned a unique ID, the from and to IDs of the nodes that the edge connects, a link type attribute explicitly defining the transport modes being connected through each edge, and a usage type column defining if the connection is used specifically for freight transport or passenger transport or both. Each multimodal connection is duplicated to create two unidirectional edges between connecting nodes, in order to specify the direction of the dependency between networks.

## Generalized construction costs for road and railway assets

In addition to data aggregation and cleaning, an essential aspect of this continental-level dataset is the establishment of potential investment costs for the upgrade and maintenance of planned railway lines and road corridors. This includes both the capital investment for the implementation and update of the infrastructure and the costs associated with operations and maintenance.

For road corridors, values of reconstruction/upgrading cost by road typology (*motorway*, *trunk*, *primary*, etc.) and condition (paved/unpaved) per km per lane are extrapolated and combined from AfDB (2014)^74^, World Bank^75^, Koks *et al*.^76^ and Integrum Construction (2021)^77^.

The IEA report^78^ provides 2010 operation and maintenance cost estimates per lane-kilometer to be used in addition to initial capital costs. These figures are adjusted and updated to reflect 2025 values considering average inflation in the continent in the considered years, ranging between five and six percent annually^79^. For railway investments, upgrading costs from Koks *et al*.^76^ and operation and maintenance costs from Dulac *et al*.^78^ are gathered and applied in the same way. Minimum, maximum and median values are used to compute the range of total investments needed to maintain the road and railways assets through 2050, assuming the initial capital investment happening in 2025, a 20-year lifetime and operation and maintenance happening every 4 years^78^. An eight percent discount rate is assumed for both the costs actualizations^80^. Table 1 reports the average values per unit of costs used for roads (see Supplementary table S3 for the values used for railways).

**Table 1:** Data on costs estimates, assembled from different sources, applied to individual road transport edges based on the road types. The costs estimates are used to infer the level of investments needed to construct, upgrade and maintain existing road assets.

Cost type	Condition	Road type	Costs (USD/km/lane) (2025 estimates)			Sources
			Median	Minimum	Maximum	
Construction and Upgrading	Paved	Motorway/Trunk	620,863	602,800	638,927	AfDB (2014)^74^,World Bank (2018)^75^, Koks *et al*.^76^, Integrum Construction (2021)^77^
		Primary/Secondary	551,981	528,562	575,400	
		Tertiary	264,372	200,124	328,621	
		Bridge	1,301,500	976,125	1,626,875	
	Unpaved	Primary	21,962	20,595	23,330	
		Secondary/Tertiary	18,317	17,498	19,138	
		Bridge	21,962	20,595	23,330	
Capital	Paved	Motorway/Trunk/Primary	2,160,000	2,087,557	2,232,443	Dulac *et al*.^78^
Reconstruction/Upgrade			360,000	347,926	372,074	
O&M			63,000	60,887	65,113	

These cost estimates are approximations derived from actual project expenses that can vary due to multiple factors, including country-specific and location-specific conditions, inflation rates, and the discount rate applied for cost adjustments.

## Data Records

The spatially explicit, harmonized AfTS-Db is publicly available and can be explored as a dataset record at Zenodo^81^ (https://zenodo.org/uploads/17861120). These files can be easily accessed, visualized, and manipulated using standard GIS applications such as QGIS or ArcGIS. We visualize and discuss interesting attributes of the data below.

The database includes: (1) Individual Geopackage (gpkg) files for railways, roads, maritime, inland waterway (IWW) and airlines networks. The files are named as africa_{mode}_network.gpkg, where mode = {railways, roads, maritime, iww, and airport}. Each file contains two layers (nodes and edges) corresponding to point and line feature datasets respectively with associated attributes. This format is preferred as widely supported in GIS software. (2) A Geopackage (gpkg) file called africa_multimodal.gpkg which only contains the multimodal edge connections between different transport modes. (3) Investment costs tables (as.csv files) for main road corridors only and railways lines. These files are named africa_corridors_costs.csv and africa_rails_costs.csv respectively (4) A metadata table Excel file (METADATA.xlsx) describing each component dataset of the database, with details of each column. Additionally, for completeness, the OurAirport nodes dataset for Africa is present as geopackage (gpkg) file called africa_airport_ourairport.gpkg.

Table 2 shows the list and description of the common column names with attributes available in the dataset, additional description is available in the METADATA.xlsx file available with the database. The database comprises five primary transport mode types: roads, railways, maritime routes, inland waterways, airports. These are joined by multimodal edges, which are notional lines. To ensure systematic and standardized data compilation, the common columns establish uniform guidelines for data classification, attribution, and integration across different transport modes. This approach ensures clarity, traceability, and interoperability across various networks and allows for the creation of multimodal connections.

**Table 2:** List and description of the harmonized column names and their attributes found in every type of nodes and edges infrastructure layers in database.

	Column Name	Structure	Content
NODES	id	{Mode type}n_[0/…/n]	Identification code of the point
	iso3	ISO 3166 Alpha-3 code of the country	Country code
	component	Number	Identifier for all nodes belonging to the same connected subgraph cluster within a network
	source	Modelled/[specific source(s)]	source(s) of the component of the dataset
	geometry	POINT	Point geometry of nodes
**EDGES**	id	{Mode type}e_[0/…/n]	Identification code of the edge
	from_id	{Mode type}n_[0/…/n]	Identification code of the initial point
	to_id	{Mode type}n_[0/…/n]	Identification code of the final point
	from_iso3	ISO 3166 Alpha-3 code of the country	Country of the initial point
	to_iso3	ISO 3166 Alpha-3 code of the country	Country of the final point
	component	Number	Identifier for all edges belonging to the same connected subgraph cluster within a network
	source	Modelled/[specific source(s)]	source(s) of the component of the dataset
	geometry	LINESTRING	Line geometry of edges

Mode type: {iww = inland waterway, port = maritime port, maritimeroute = maritime route, rail = railway, africa-latest = road, airport = airport, intermodal = multimodal}.

## Data Overview

### Rail development and connectivity in Africa

**Fig. 2:**
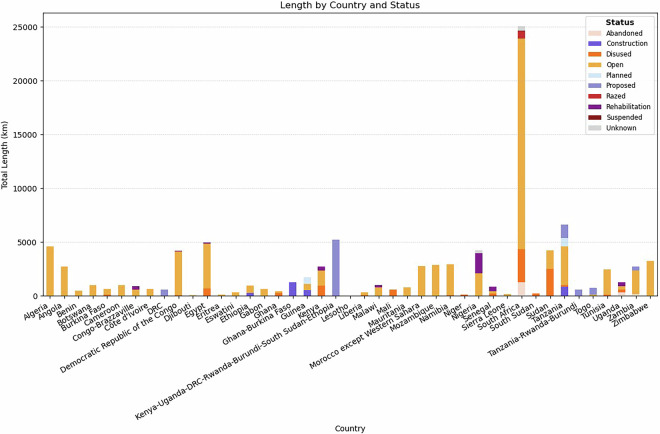
Estimates of total length (values in km) of railways lines by country and known or inferred operational status.

**Fig. 3:**
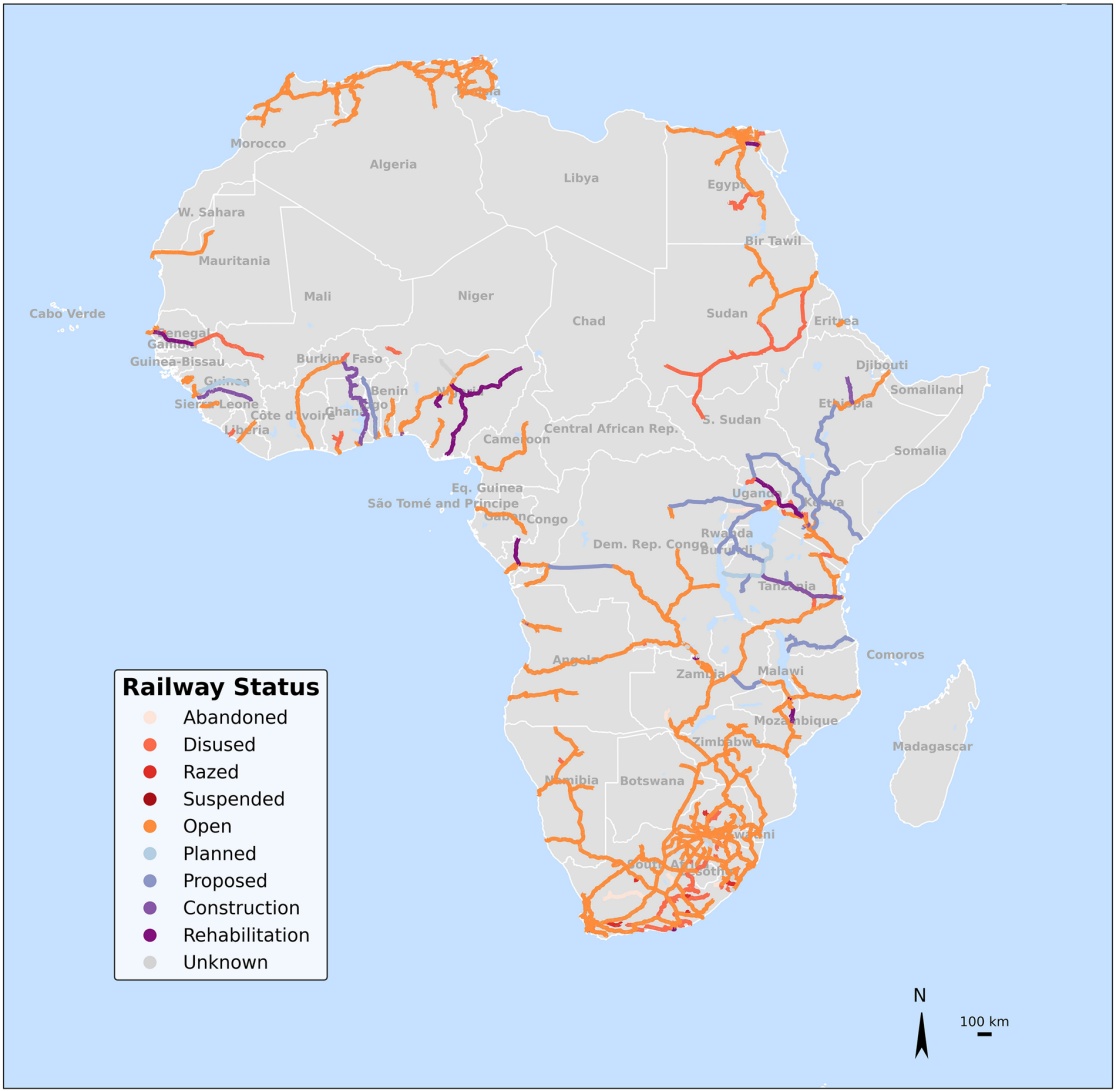
Map representation of the railway network of Africa with the line highlighted by their known or inferred operational status. The map prominently shows the extent to which the network is “Open” and the new routes which are being “Planned” or”Proposed” or under “Construction” and “Rehabilitation”.

With a total of 99,373 km of railway lines in our database, Africa’s rail network has primarily been developed to enable connectivity of important locations of economic activity (e.g. mines) towards ports. The database includes 986 railways corridor routes, differentiated by line names. Railway lines are also classified into nine different status categories: *abandoned, under construction, disused, open, planned, proposed, razed, under rehabilitation, and suspended*. Figure 2 presents railway line lengths by country and some proposed transboundary projects, providing a sense of the condition and status of development of the railway network across Africa. Figure 3 provides similar information spatially, which also gives a sense of the connectivity and integration of the railway network in Africa. Planned projects are those which have been approved and seem to be ready for construction in the near future, while proposed ones are still in the stage of feasibility assessments at the time of writing. Furthermore, the spatial accuracy of some proposed projects in the database might change, since their information was derived by digitizing maps from reports. Additionally, some lines are marked as unknown when this information was unavailable in any of the consulted sources (see Fig. 3).

The majority of the railway network in Africa is labelled as open (70% of the total length), while 10% is disused and another 10% is classified as proposed or planned. 3,651 kms are labelled as rehabilitation (4% of the total length), while the rest is either abandoned, razed, suspended or unknown (4% in total). The southern part of the continent has the highest concentration and connectivity of rail lines, led by South Africa’s dense rail network that is used extensively for coal and metal transport^82^. There are under construction, planned, proposed and rehabilitation projects as part of the East Africa railway plan to create an integrated railway network across Kenya, Tanzania, Uganda, Burundi, Rwanda, South Sudan, Ethiopia and Democratic Republic of Congo (DRC)^83^. In Western Africa, where large part of the railways is undergoing rehabilitation or construction, the network is less integrated across countries, due to financial constraints and limited political initiative^84^. An additional consideration for inferring connectivity of the rail network is the gauge width of the lines, which indicate the types of trains that can operate between different network corridors. The database shows that most of the existing railway lines in Africa are narrow gauge (1067 mm width or lower), while new construction, planned and proposed projects are being developed to standard gauge (1435 mm width) (see Supplementary Fig. S1). This has created widely-discussed challenges regarding the costs of development and lack of integration of the East Africa networks with those going towards the central and southern parts of the continent, which are narrow (meter) gauge^85^.

### Road network connectivity and corridor development in Africa

The dataset reports a total of 1,004,512 km of roads, classified here (for ease of discussion) into five broad highway typologies - *primary, secondary, motorway, trunk, and other* (which includes *tertiary, living street, residential, service, track, construction and unclassified*). A map representing road typologies is available in supplementary material Fig. S2. The majority of the roads in the dataset are classified as secondary roads (39% of the total length), followed by primary and trunk roads respectively representing 27% and 20% of the total length. Tertiary and motorways comprise 4% and 3% of the data, and the others (7%) make up the rest of the dataset. Tertiary and other roads are included since they connect these main routes to key points of interest, such as stations, ports, and airports, ensuring accessibility to areas not reached by larger roads.

For road infrastructure, the dataset highlights 41 key transport corridors, primarily sourced from the African Development Bank (AfDB)^8,26^. These corridors play a crucial role in facilitating regional and continental connectivity, with several overlapping segments that demonstrate the interconnected nature of Africa’s transport network. Transnational corridors are particularly significant as they provide efficient routes for both passenger and freight transport across multiple countries. Their expansion and modernization represent a strategic investment in the continent’s future, improving market access, reducing transport costs, and fostering economic opportunities in both urban and rural areas.

**Fig. 4:**
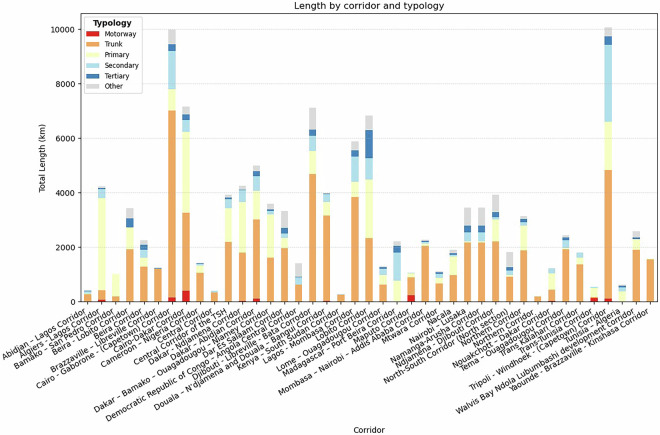
Total length (values in km) of major roads corridors in Africa by typology of road classifications as defined by OpenStreetMap (OSM). The extent to which the corridor roads are classified as “Motorway” or “Trunk” can be considered indicative of the quality of roads along the corridor.

Figure 4 illustrates the total length of named road corridors stacked by highway typology. Most sections of these corridors are trunk roads and some are motorways, which are generally in good condition^86^. The stacked bar plot highlights significant sections classified as primary, secondary, tertiary or other, which is generally not in good condition. The data shows that majority of the roads on these corridors at the moment are paved (71%) but there is room for improvement of these infrastructures to lead development, since 29% are currently unpaved. This aligns with other studies that have indicated that work is still required to upgrade these roads and improve the access of people to high quality paved roads which is far lower in Africa compared to the rest of the world^19,87^.

**Fig. 5:**
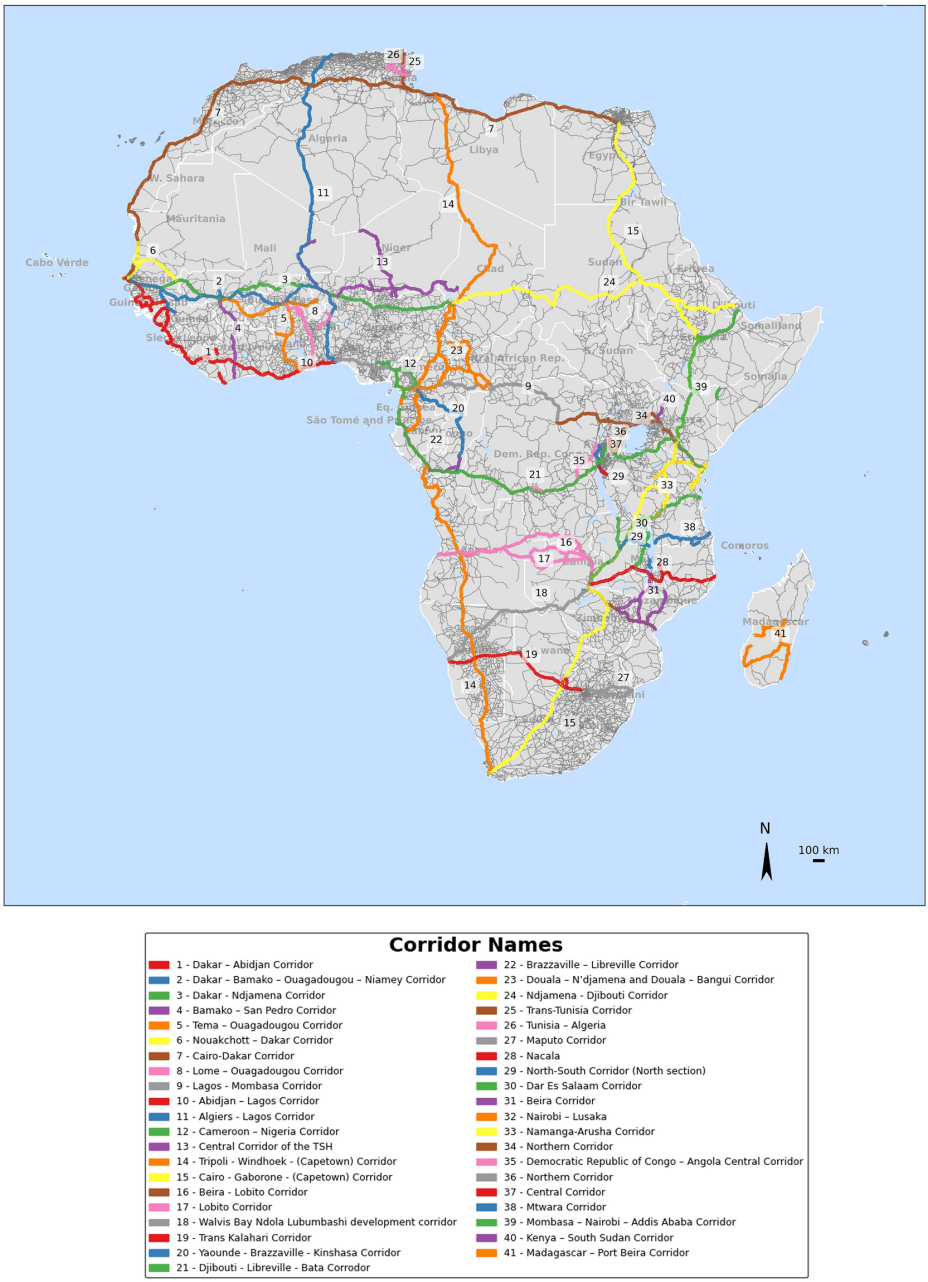
Map of the road network created in the database, highlighting major corridors in Africa identified from reports by AfDB^8,26^.

The map in Fig. 5 shows the extent of the integration and connectivity being achieved through different corridors in Africa. Significant corridors like the Cairo-Gaborone-Cape Town (15) and the Tripoli-Windhoek-Cape Town corridor (14) connect across the north-south length of the continent, and overlap with a few other regional corridors. Similarly, east-west connectivity in the continent is facilitated via the Cairo-Dakar corridor (7), Dakar-Djibouti corridor via N’Djamena (3 and 24), Lagos-Mombasa corridor (9), Lobito corridor (16 and 17), Walvis Bay corridors (18 and 19).

### Waterways network connectivity and maritime port capacity in Africa

**Fig. 6:**
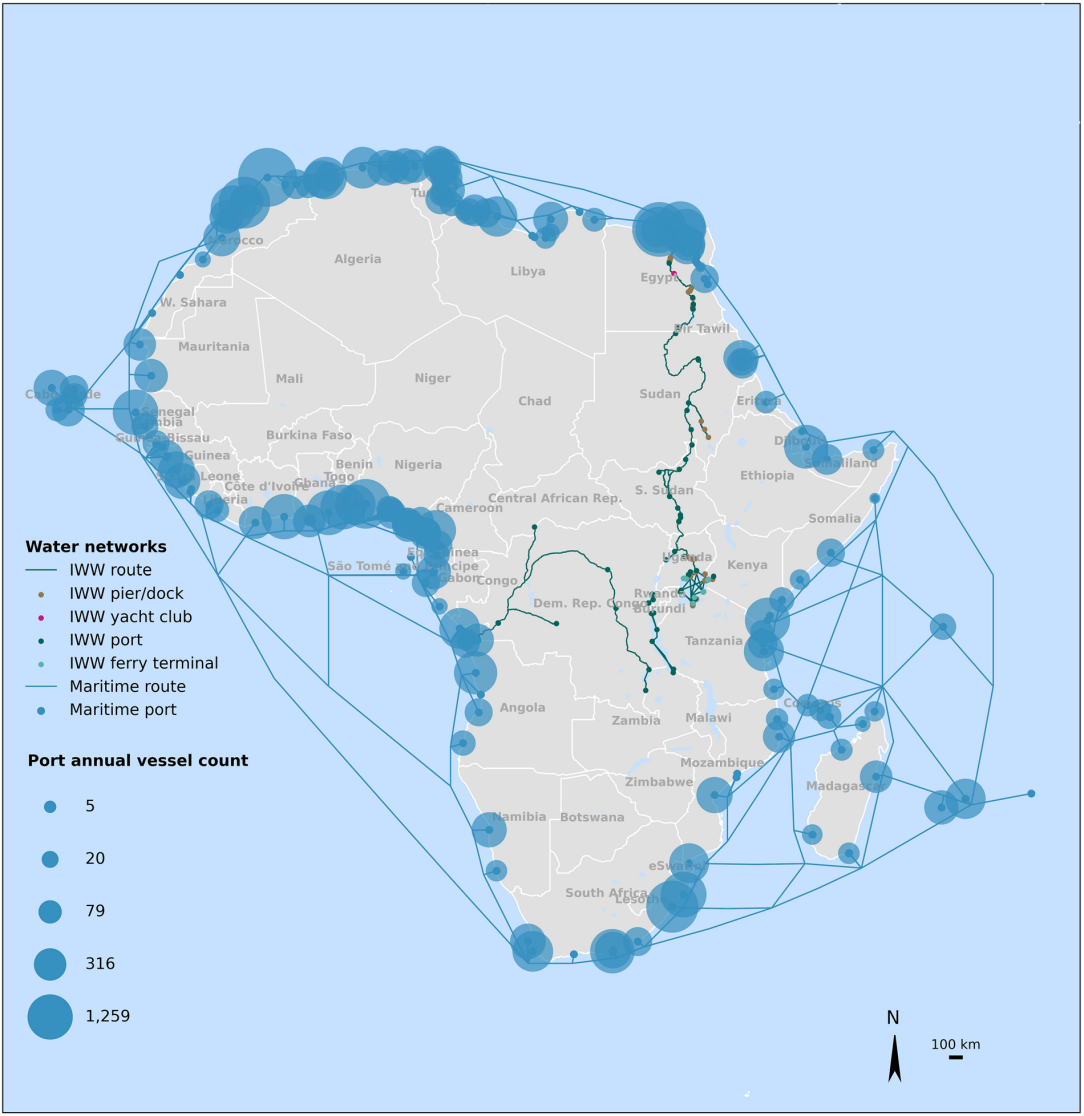
Map representation of locations of maritime ports and inland nodes (ports, pier/docks, yacht clubs ferry terminals) and shipping routes between ports of Africa, created in the database. The sizes of the maritime ports in terms of total annual vessel counts across all vessel types handled at ports are also highlighted (smaller dots - smaller number of vessels, bigger dots - bigger number of vessels), based on the observed shipping data compiled by Verschuur *et al*.^59^ and Port Watch (2025)^60^.

The database features a total of 179 major maritime ports spread across the continent, 60 inland ports on Lake Victoria, Lake Tanganyika, Lake Kivu (Rwanda), the Congo and Nile rivers, and 72 docking points (piers/docks, landing sites, yacht clubs) along the Nile river. Figure 6 displays them on a map, together with the inland and maritime shipping routes between ports of Africa used in the network. The map also shows the annual number of vessels called (arriving and departing) at maritime ports visually represented on the map using bubble sizes, where the bubble size reflects the total annual vessel count per year. The number of vessels called at a port is a good proxy of port size in terms of handled cargo numbers and volumes. For instance, previous research showed how the total container vessel tons called at ports in Africa corresponds well with the number of containers handled^88^.

Though sparsely located and used, inland ports and waterways in Africa serve as interconnectors along corridors. Ports on Lake Victoria and Tanganyika are part of the integrated (but often underutilized) corridors connecting the ports of Dar-es-Salaam and Mombasa to the landlocked countries of Uganda, Rwanda, Burundi, and Zambia and also to the eastern parts of DRC^89,90^. This is also evident from the map of the railway network in Fig. 3, which shows some railway lines extending towards the lakes, where they connect to the inland ports. Inland waterways along the Congo river are widely used as the main transport mode, especially in DRC where good quality road and railway networks are limited^65^. As Fig. 6, the inland waterways also connect with the maritime ports on the western coast towards Congo. The inland network along the Nile river has the potential to revitalize the inland transport corridor from Lake Victoria ports in Uganda and Tanzania towards the maritime ports of Alexandria and Damietta in Egypt^63^. The Nile inland waterways were once used extensively for goods transport, but their modal share has declined considerably due to lack of infrastructure development and maintenance, compounded by the shift toward the improved road corridors^63,91,92^. Currently the AU-PIDA is supporting a US$ 1.25 billion initiative to develop and start operations along this corridor by 2042^93^.

Maritime port hubs are scattered quite uniformly along the coast, as is shown in Fig. 6. However, the density of ports is higher in the North and West of the continent compared to the South and the East. These hubs serve as gateways for 16 landlocked countries where about 30% of the population of the continent reside^94^. Some notable port hubs in Africa include: (1) Tangiers (Morocco), Demiatte and Said (Egypt) in North Africa; (2) Dar es Salaam (Tanzania), Mombasa (Kenya) and Maputo (Mozambique) in East Africa; (3) Durban and Cape Town in Southern Africa; (4) Walvis Bay (Namibia), Lobito (Angola), Lagos (Nigeria), Abidjan (Cote d’Ivoire), Tema (Ghana) and Dakar (Senegal) in West Africa. While there are a several ports across the continent, most of them lack cargo handling and operational capacity and connectivity to hinterland^95^, prompting the need for an integrated approach to understanding the linkages across ports and different land corridors^19^. The routes between African ports, also compiled in our database and highlighted in Fig. 6, helps understand the transshipment opportunities in Africa that would enable clusters of ports to handle more cargo efficiently^62^.

### Airport usage and distribution in Africa

**Fig. 7:**
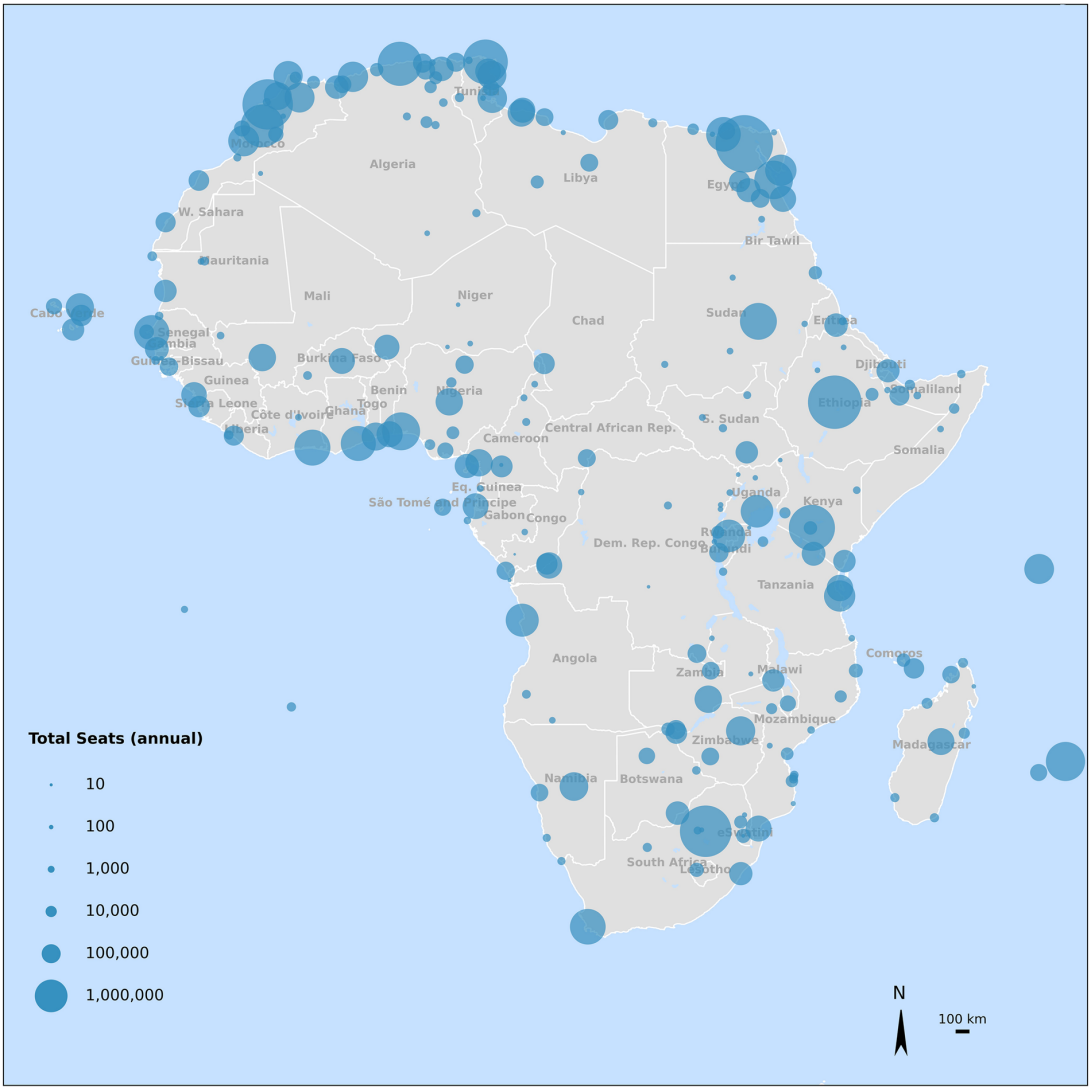
Map representation of locations of 234 African airports in the database classified by annual number of seats at airports (smaller dots - smaller airports, bigger dots - bigger airports). The data on airport usage is obtained from the World Bank^57^.

The database includes 234 airports, as shown in Fig. 7, most of them located in the largest northern countries - Algeria, Morocco, Egypt, and Mozambique - each having more than 10 airports. The dataset also records the number of available seats per airport (a proxy for annual passenger traffic numbers), visualized through a bubble representation on the map: smaller bubbles indicate lower passenger traffic, while bigger ones represent the highest. This reveals that the countries with the most airports do not always correspond to those with the highest passenger traffic, with Egypt, Morocco, South Africa, Ethiopia, and Tunisia ranking as the most served.

### Multimodal connections in Africa

In total, seven types of connections between transport nodes are identified: *airport-railway*, *airport-road*, *inland waterway (IWW)-railway*, *IWW-road*, *maritime port-railway*, *maritime port-road*, and *railway-road*. The majority of these connections link railway lines to road networks, followed by airport-road and port-road connections. The data shows that about 84.2% of all rail stations (3,715 out of 4,412) are assumed to be connected to a road link, with 72% (3,408 out of 4,412) used for passenger interchanges and 28% (307 out of 4,412) used for freight transfers. Almost all maritime ports (175 out of 179) are connected to roads, excluding some ports in small remote islands where the road network data was not included. About 31% (55 out of 179) of maritime ports are connected to railways. Similarly, all inland waterway ports and docking sites (132 out of 132) have road connections, while only 20% (12 out of 60) inland ports have rail connections. In the case of airports 234 out of 234 airports are connected to road, whereas only 2.6% (6 out of 234) airport-rail connections are identified in the data. The data highlights the poor connectivity of railways with other modes, which aligns with evidence that railways in Africa over the last decades have lost traffic to roads at major ports due to poor infrastructure quality and maintenance^96^. The data also highlights the opportunity to improve multimodal connectivity between railways and other modes.

### Investment costs for African rail and road corridors

The estimated costs related to the upgrading (investments in improvement of such connections together with operation and maintenance) of currently existing and planned road corridors as well as the ones for railways that are not yet labelled as ‘open’, represent a way to quantify how much capital would be needed from now (2025) through 2050 if an investment in improving those networks was made. The total median annual investment into upgrading and operation and maintenance costs in railways would require 23.9 billion US$/year (minimum 14.3 billion US$/year, maximum 25.6 billion US$/year), while for the 41 road corridors a total of 8.6 billion US$/year would be needed (ranging between 8.3 and 8.9 billion US$/year). These values are considered reasonable compared to the 35–47 billion US$/year of investment needed for road, railway, ports and airports infrastructures in the continent as reported by AUDA-NEPAD^97^. Of the total costs, the annual capital costs are 5.4 billion US$/year (minimum 5.2 billion US$/year, maximum 5.6 billion US$/year), in line with the investments made in road corridors between 2004 and 2022 by the AfDB^8^ (13.5 billion US$ for some fractions (18,022 km) of the 25 corridors reported) and with the 4.6 billion US$/year reported by Cervigni *et al*.^98^.

## Technical Validation

### Position accuracy of node locations

The database created in our study integrates multiple independently validated sources. The initial validation involves a check of the position accuracy of the nodes (airports, ports, railways stations) and edges (road segments, railway lines). We note that we are representing large assets like airports and ports as point locations, which means that “position accuracy” here refers to the geolocated point (xy-coordinate) being within the spatial footprint of the asset and attributed to a main area such as an airport terminal building or a port dock. For airports the OurAirports dataset that we have used has been found to have more than 91% positional accuracy globally when compared to satellite imagery, and the positional accuracy is close to 100% for large and medium sized airports^99,100^. We note that in the OurAirports data the geolocation of the airport nodes in most cases is on the runways, which we corrected manually through comparison with Google satellite imagery, to make sure that the points correspond to the main airport terminals. We also found the original OurAirports data to be useful to be kept as is as an additional layer for geolocating runways for the airports dataset; and hence we have also kept and reported this data, while making reference to the original OurAirports source for location accuracy. The positional accuracy of our selected airports is 100% when aligned with Google Satellite imagery. For inland waterway ports we have 132 locations of interest (ports and docking sites) in our data, where we are also able to manually check and geolocate accurately with Google satellite imagery. Hence, the positional accuracy of inland ports is also 100% in the data. For maritime ports, the global datasets from Verschuur *et al*.^59^ (and its updated version in PortWatch^60^) contribute 79% of the ports (142 out of 179) and are based on extracting locations from satellite images with very high position accuracy. The remaining 21% maritime ports (37 out of 179) in our data are from USGS^51^ and Thorn *et al*.^28^ which again have high position accuracy. We again checked these ports with Google Satellite imagery and were able to improve the position accuracy to 100%, where there were any inconsistencies.

The roads and railways datasets are created from OpenStreetMap (OSM), which is widely recognized, extensively used and updated constantly to reflect new developments. Globally, including countries in Africa, OSM roads dataset for major roads (motorways, trunk, primary and secondary) has been found to be more than 80% complete^101^. OSM roads datasets also have been shown to have high position accuracy and better spatial coverage than official road datasets in Africa^102,103^. The coverage and positional accuracy of OSM railways is also very complete^104^, and has been enhanced by cross checking with country reports and known sources of new railway projects in Africa (see Supplementary Table S2). Additional data sources have been incorporated, including Thorn *et al*.^28^– a key reference on Africa’s new corridor projects. Critical infrastructure data such as road corridors, ports, railways, and airports have been meticulously compiled from authoritative sources, including AfDB, AUDA-NEPAD platform, and official national and regional master plans and reports (see Supplementary Table S2).

### Accuracy of tagging important railway stations

A key aspect of the railway dataset is the identification and tagging of important stations as facilities associated with different activities. In Africa most railway infrastructure has been built to connect to locations of economic activity and facilitate transport of resources towards ports. For example, our railway dataset contains about 100 stations tagged as named mining facilities – which means they are stations linked to mines. In total out of 6,045 named railway nodes (important stations, stops, halts) in our dataset 468 nodes have an additional tag of the type of facility they serve (see Supplementary Fig. S3).

The accuracy of the tagging of facilities is verified with cross checking these locations with multiple sources and finding the straight-line distance between the rail facility and the facility identified from the other sources. Cross checking is done with: (1) Google Maps, via an API search based on the name and the location of each facility to see if it exists on Google Maps and compare the location of the railway facility with geotagged information from Google Maps; (2) Global remote sensed mining footprints^105^ to infer if railway stations tagged to serve mines are actually in proximity of mines; (3) Location information on mineral processing plants, oil depots from the USGS database^51^ to confirm if the railway stations are serving such facilities; (4) The airports, ports (maritime and inland) locations assembled in this study to confirm that railway station tagged to serve such locations are close to them; and (5) Sense checking locations with Google satellite imagery to confirm that they are correctly tagged (e.g. a food depot or freight depot can be found next to the station facility tagged to serve such locations).

**Fig. 8:**
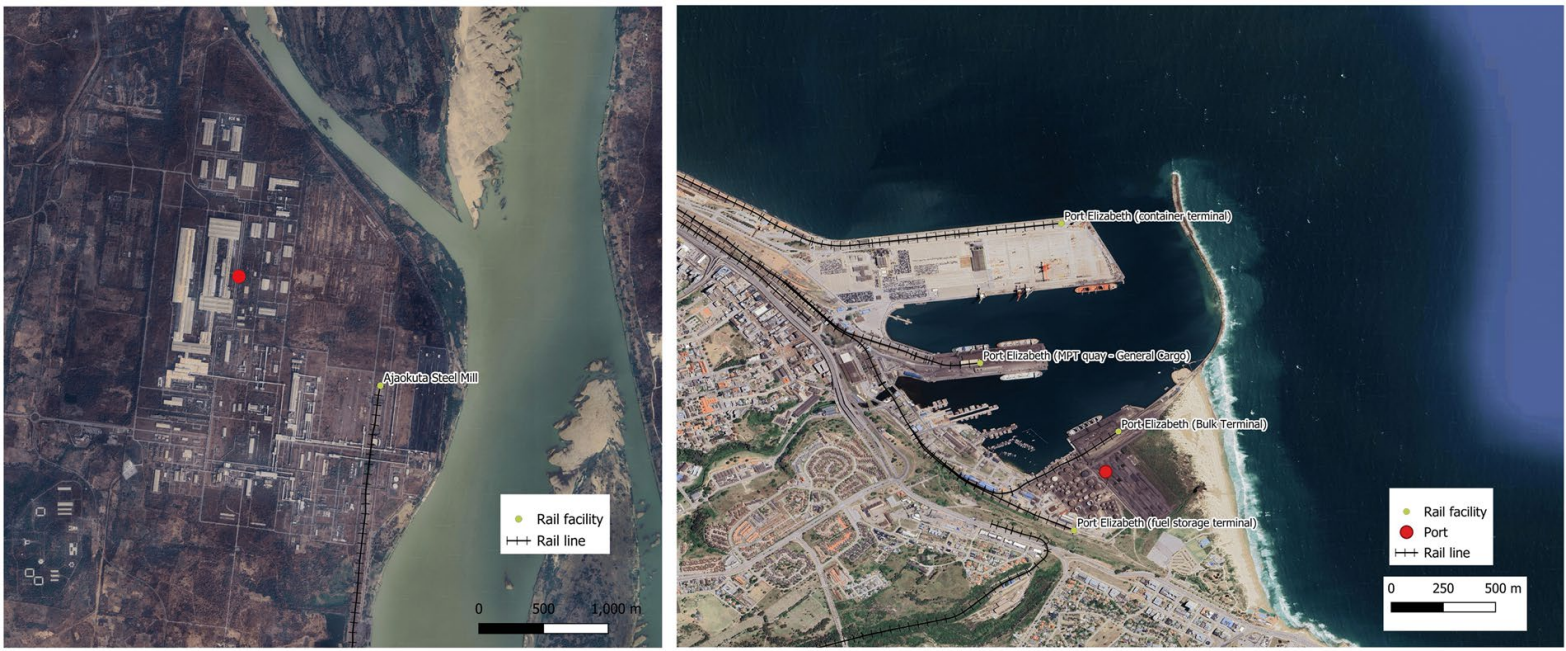
Location specific validation of rail facilities created in the database. The left map shows a Steel Mill in Nigeria (identified by the Google Map tag shown as the red dot) and the rail facility connected to it. The right map shows Port Elizabeth in South Africa (located in the port dataset by the red dot) and multiple railway facilities serving the port. Both these examples highlight how location proximity is the effect of approximating large establishments in single point locations.

The verification checks show that 27.6% of rail facilities align exactly with the similar locations inferred from other data sources and 93.4% are within the 1 km distance range (See Supplementary Fig. S4). In instances where rail stations serve airports, ports or large manufacturing sites the estimated distance proximity increases because point locations from the other sources (Google Maps, Ports or Airports dataset) are also approximated over large areas. Examples of this are shown in Fig. 8 where the left figure shows the identified rail station for a steel mill in Nigeria and the corresponding Google location tag which is more than 1 km away, and the right figure shows several rail container terminal facilities serving Port Elizabeth in South Africa whose point location is tagged to be about as far as 1.7 km away from the farthest rail terminal. Such examples demonstrate that the rail location tagging is very accurate but due to approximations of large facilities as point locations it might seem that they are very far away from the facilities they are meant to serve.

#### Multimodal links evaluation

To validate the representation of multi-modal connections, we have measured the straight-line distance between the assets connected via each multi-modal link. The histogram of the lengths of the multimodal links (see Supplementary Fig. S5), which is an indicator of the proximity of connected inter-modal assets, shows that 61.9% of links are less 100 m long and 97.6% are less than 1 km long. The links connecting different modes to roads are generally of shortest lengths, given that every port or airport or railway station have local access roads. Multimodal links of lengths greater than 1 km might seem unrealistic but these represent known connections between know railway facilities and other assets.

**Fig. 9:**
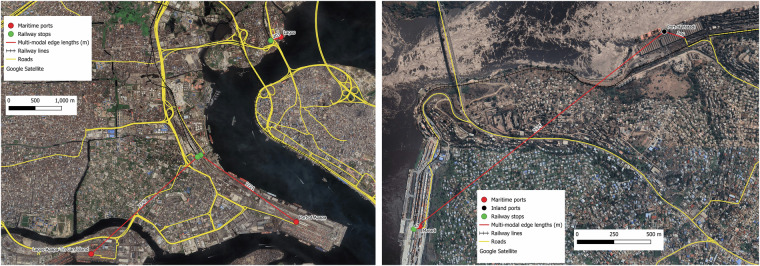
Location specific validation of multimodal connections created in the database, by zoomed in manual checking of satellite imagery of locations of interest. The left map shows the different terminals (red dots) at the Port of Lagos in Nigeria, whose connectivity of the rail stations (in green) is inferred through the multimodal edges (red lines). The right map shows the Inland Port (black dot) and the Maritime Port (red dot) of Matadi in Democratic Republic of Congo, showing both ports connected to the rail terminal which comes in the maritime port dock.

Two examples in Fig. 9, show the visual process to validate the longest multimodal edges. The left figure shows the connectivity between rail stations and different terminals at the Port of Lagos in Nigeria. Two of the rail stations are very far away (in excess of 2.2 km by station line distance) from the port terminals, but we know that they bring goods in and out of the port, which is also spread over a very large area (as visible from the figure). Hence, the rail stations are connected to the port via the multimodal edges (red lines). The right figure shows a similar issue at the Port of Matadi in DRC, where the inland terminal and the maritime terminals (spread over large areas) are both served by the same rail station, which is connected to both. One of these connections is in excess of 2.1 km. As already stated, though multiple roads leading to the port and rail connection are visible in the validation process, only the closest ones are chosen due to the point simplification of the ports location (this assumption is especially visible in the left map image).

#### Comparison of rail and road network lengths

**Fig. 10:**
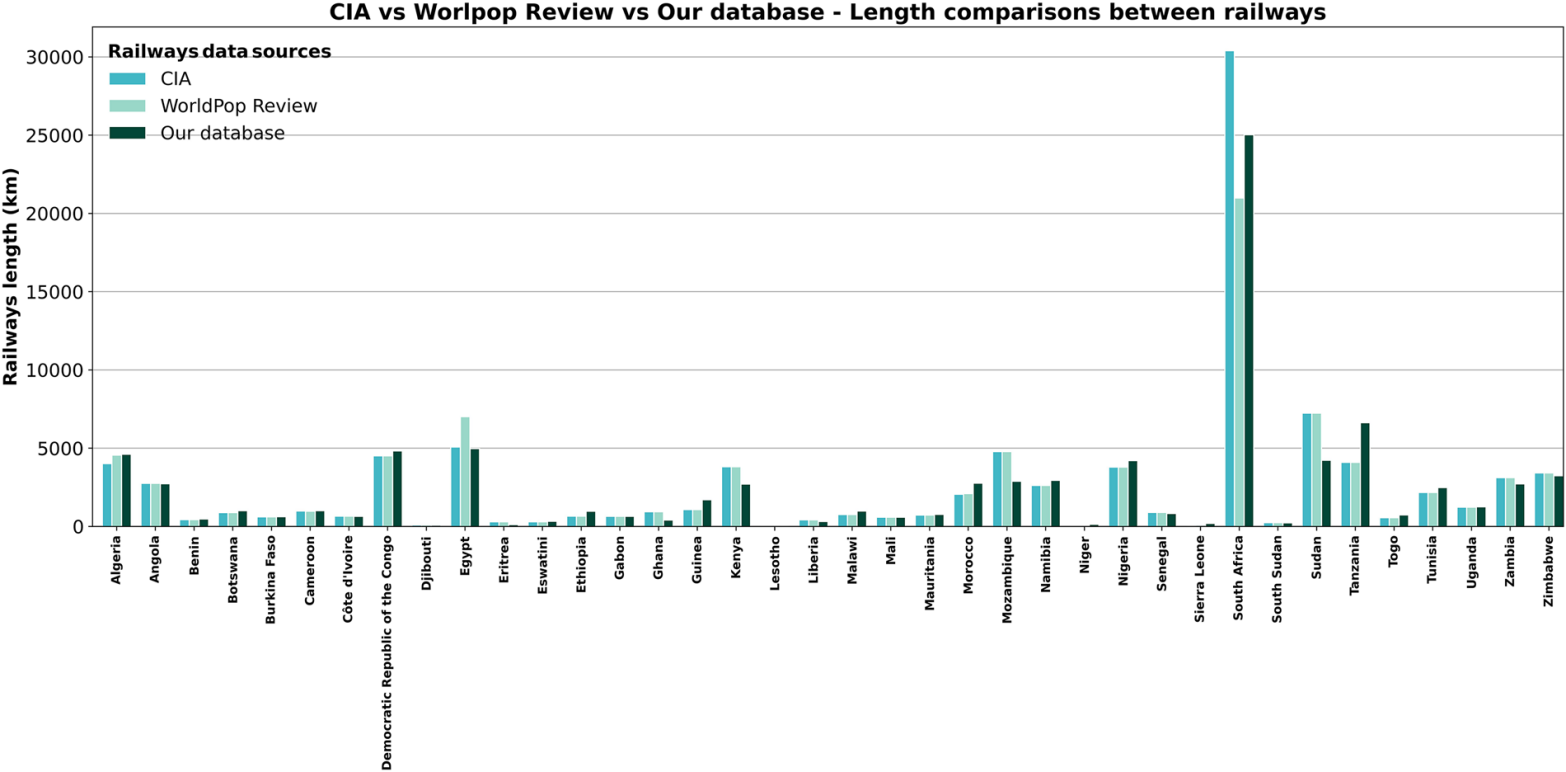
Comparison of our railway network database length, in dark green, and the length by country of CIA and World Population Review values (blue and light green respectively).

The lengths of railways are compared for completeness to national values obtained from the Central Intelligence Agency (CIA) factbook^106^ and World Population Review^107^ values, as reported in Fig. 10. The total length difference is −6% and 1% with respect to CIA and World Population Reviews, respectively, which shows a very good agreement of our dataset with other known datasets. For Eritrea, Ghana, Sudan, Mozambique, Kenya, Zambia, Zimbabwe, and Egypt our estimated lengths are lower than both datasets, which could be because these datasets contain information of abandoned railway lines which we do not have. For example, in Eritrea we only include an estimated 122 kms of operation network in our data, whereas the other data sources report 306 kms of rail lines most of which were in the 19^th^ century and have been abandoned^108^. On the other hand, in Tanzania and Nigeria our dataset contains some proposed and planned project, which are not reflected in other databases. Cote d’Ivoire, Angola, Gabon, Mali, Uganda and Burkina Faso lengths are very similar across all datasets, with differences between −2 and +2% (see supplementary material Table S4). The magnitude of total length per country is also coherent within the datasets, with South Africa, Sudan, Egypt having the highest values in all of them, and Lesotho and Niger containing the least length of railway network.

For roads there are no available open-source alternative data sources that can be easily compared with OSM. While the CIA handbook also estimates total paved and unpaved road kms by country, it cannot be compared with our database because the CIA handbook data is from different years and provides no information on how the lengths of paved and unpaved roads in each country were estimated and which roads were considered. Other known open-data resources for roads include: (1) The Global Roads Open Access Data Set (gROADS)^109^, which contains road information between 1998–2010 only; (2) Global Roads Inventory Project (GRIP)^110^, which was last updated in 2018 and has different typologies for roads than OSM making it difficult to compare the two datasets; (3) Overture Maps^111^, which is a new road product built on OSM data.

**Fig. 11:**
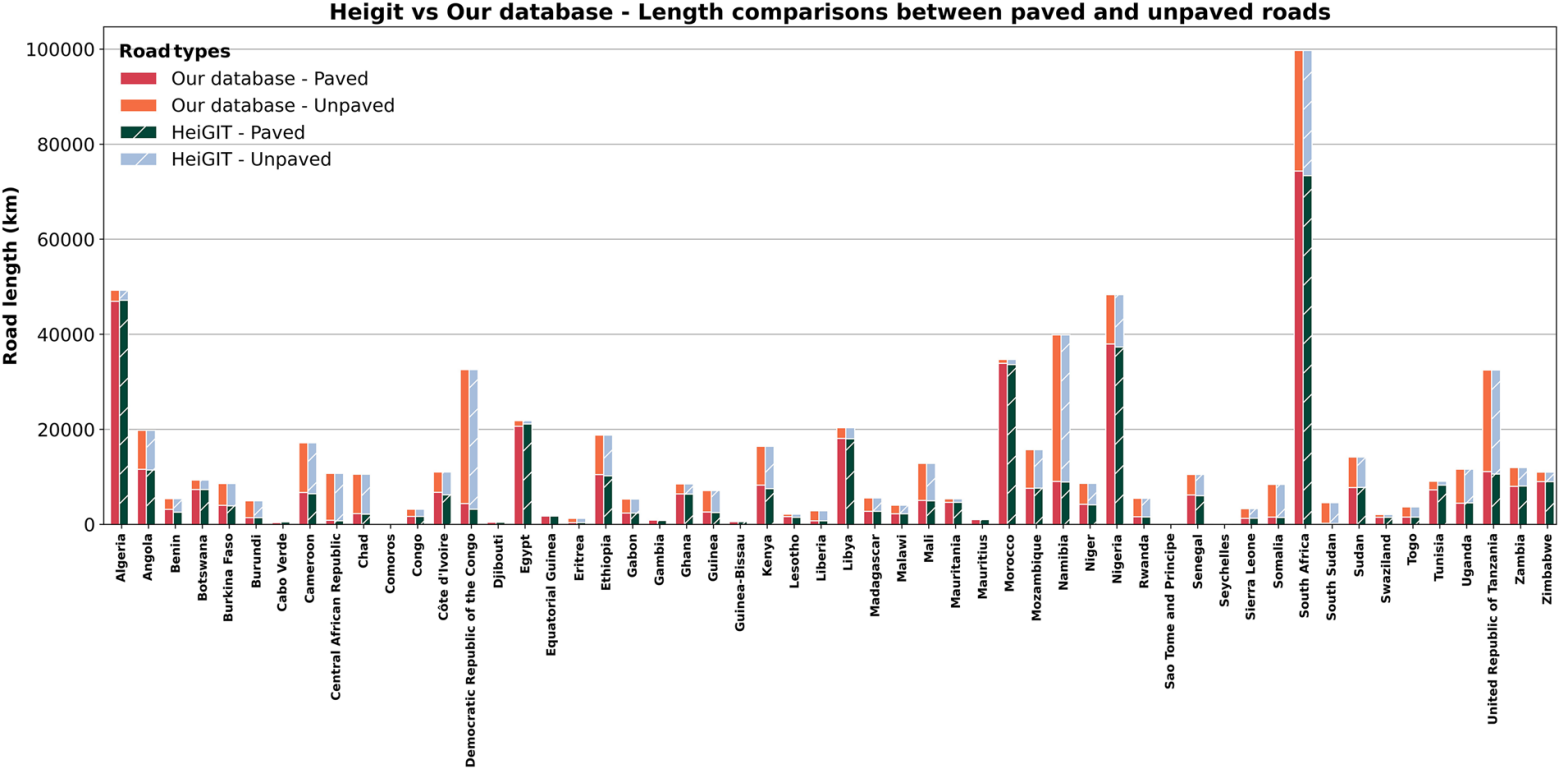
Comparison of our roads network database paved and unpaved lengths, with the HeiGIT data.

A suitable validation of our OSM data would be to get a sense of the assigned length of paved and unpaved roads and compare them with other datasets. We estimate that roughly 75% of lengths of roads on main corridors across Africa are paved, which aligns with a previous study of Africa-wide assessment of pavement conditions^87^. As shown above (see Section on investment costs), our estimates of cost requirements for corridor road upgrades and maintenance align with those of AfDB, which are built on our assumptions on paved and unpaved roads. At a more spatial level, a recent initiative from the Heidelberg Institute for Geoinformation Technology (HeiGIT) integrates OSM road datasets globally with street-view imagery to improve identification and prediction of paved and unpaved roads^112^. For Africa the HeiGIT database adds information on road conditions (paved or unpaved) to roughly 660,000 kilometers of roads not tagged in OSM. We compare the lengths of our estimated paved and unpaved roads with the HeiGIT data, where the OSM road IDs are common. We note that that due to regular updates in OSM not all IDs between our dataset and HeiGIT are common, and we also notice that in the HeiGIT data some road segments do not have any OSM IDs. As shown in Fig. 11 below, there is a good overall agreement between the common roads in the two datasets, with total lengths of our paved roads being 0.01% more than those estimated by the HeiGIT data (418,248 km vs 412,938 km) and the total lengths of the unpaved road being 0.02% less (279,369 km vs 284,678 km). There are some differences at the country level roads networks, as presented in Supplementary Fig. S6 and Table S5. For example, for a smaller island such as Capo Verde we are overestimating the lengths of unpaved roads for 80%, but these are very small lengths which probably include one or two roads. We also notice that we are overestimating the lengths of unpaved roads in countries such as Egypt, Tunisia, Libya and Algeria where most secondary roads are paved, whereas in our assumption these are considered unpaved. We note that these are a small proportion of the overall networks as well. In countries such as Benin, Central African Republic, Comoros, DRC, South Sudan we are overestimating the paved roads in comparison to HeiGIT because of the assumption that primary roads are paved, whereas they might not be paved in most of their countries. Again, these are small proportions of the country’s overall network. Generally, road conditions correlate well with the Human Development Index (HDI) of countries, which is evident from the HeiGIT data^112^ and hence a potential improvement in our data could be made to assume that countries with high HDI would have paved roads till the secondary level and countries with low HDI would have unpaved roads from the primary level.

## Usage Notes

The African Transport System Database (AfTS-Db) database presented in this paper is the first comprehensive continent-wide dataset integrating multiple transport networks of roads, railways, aviation, maritime and inland waterways across Africa in a standardized format and creating a unique multi-modal network-of-networks product. It combines several well-known data resources and improves upon their quality by enhancing spatial accuracy of point assets, adding connectivity within and across networks, and introducing a richer understanding of the locations of key inter-modal assets and the extents of key corridors across Africa.

The database relies on open-source datasets such as OSM, PortWatch, OurAirports, that are updated at regular frequencies. During the creation of this database we have also created Python workflows and codes to operationalize the downloading, integration, cleaning and standardization of different datasets. The Python workflows and the database are all openly available and shared along with this paper. We envision that this database can now be used as a basis for further updates and enhancements as new infrastructure developments happen across Africa. While this database provides good coverage of key locations of important inter-modal node assets (e.g. ports, airports and rail stations), we cannot claim that it includes every major inter-modal location across Africa. In general, there is a gap in information of inland waterway networks and their usage across Africa, because of the limited usage of this mode of transport. Hence, further such locations can be added in the format as prescribed in the database and improve the information quality of dataset such as OSM. Some more improvements that could also be made in the geospatial database include representing large assets like airports, ports and large railway stations (e.g. container terminals) as polygons rather than points. We note that OurAirports data already provides airport polygon, but there is no such data on ports and railway stations at large scale.

The AfTS-Db is an essential resource for researchers, policymakers, and industry stakeholders, facilitating data-driven policy formulation, infrastructure development, and the enhancement of trade and mobility across the continent. As an example, the database is useful for mapping critical mineral supply chains in Africa to understand the existing multi-modal routes via which mines send their products to the gateway ports in Africa for export globally. Important railway projects, included in AfTS-Db, along the East Africa Rail Corridor^14^ and Lobito Corridor^18^ are being planned for creating new routes for critical mineral transport out of Africa. Hence, using AfTS-Db the future changes in these supply chains could be studied and their wider impacts on trade, development and the environment can be analyzed. Also, climate risks to such developments can be studied at a very high geospatial resolution using AfTS-Db.

This database can serve as an important tool for further analysis: it can play a key role in the development of African value chains, supporting strategic planning and decision-making. By providing a standardized, high-resolution representation of African transport infrastructure, this dataset supports a wide range of applications, from transport planning and infrastructure development to economic analysis and environmental and climate risk and resilience impact assessments. Additionally, the value of including estimates of investment costs lies in providing a general understanding of the potential magnitude of project-related costs as a means of investment in future development around those infrastructures. Overall, we invite the research and practitioner community to use this database for different initiatives and also help in enhancing its quality and value in terms of improving the understanding of integrated transport network development across Africa.

## Supplementary information

Supplementary Information - The African Transport Systems Database - a geospatial database of multi-modal connected networks

## Data Availability

The Africa Transport Systems Database^81^ is an open-source database that provides comprehensive and easily interpretable information on transport networks across the African continent. It is uploaded as a dataset record accessible at https://zenodo.org/uploads/17861120 and can be easily downloaded, visualized and used for analysis with any GIS Software or coding programs. This resource can be utilized to conduct various analyses and pre-assessments, contributing to evidence-based decision-making and sustainable development.

## Code availability

The different resources that were created and employed to extract and clean datasets are available through multiple GitHub repositories. Access to the codes used to clean and manipulate the final versions of the data is available and hosted under an open source MIT license at: https://github.com/nismod/Africa-transport-database^113^. This repository combines the geospatial transport networks and standardized column names. Codes to clean the railway network and add detailed country specific attributes are accessible under another open-source GitHub repository here: https://github.com/trg-rail/africa_rail_network. The codes for operationalization for downloading and creating network representations from OSM raw data, via the Open-Gira repository, are available here: https://github.com/nismod/open-gira. Further Open-Gira documentation is provided here: https://nismod.github.io/open-gira/user-guide/usage/network-creation/road.html and https://nismod.github.io/open-gira/user-guide/usage/network-creation/rail.html.
