# Supplementary material for: The African Transport Systems Database - a geospatial database of multi-modal connected networks
Source: Sci Data. 2025 Dec 24;13:166. doi: 10.1038/s41597-025-06483-7 (PMC12873337; doi:10.1038/s41597-025-06483-7)
Supplement: Supplementary file 1 — Supplementary Information - The African Transport Systems Database - a geospatial database of multi-modal connected networks [file 41597_2025_6483_MOESM1_ESM.pdf]

# Supplementary - The African Transport Systems Database - a geospatial database of multi-modal connected networks

Silvia Colombo[1], Raghav Pant[1*], Marcus Young[2], Fred Thomas[1], Tom Russell[1], Jasper Verschuur[3], Jim W. Hall[1]

1. Environmental Change Institute, University of Oxford, Oxford, UK
2. Transportation Research Group, University of Southampton, UK
3. Faculty of Technology, Policy and Management, Delft University Technology, Delft, Netherlands

*Table S1 Available literature and databases on transport infrastructures of the Continent.*

| Source | Data on Transport infrastructures | Gaps |
|---|---|---|
| UNCTADstat database [26] | Trade and transport statistics for African countries, including multimodal freight movements. | Statistical data by Country on transported freight, no geographical database. |
| Mphigalale (2020) [15] | Qualitative and quantitative analysis of infrastructure investment in Angola, DR Congo, and Ghana. | Not continental level, not geographical. |
| Obeng et al. (2022) [18] | Assessment of Ghana's railway transport condition. | Focus limited to Ghana's railway system. |
| Ogochukwu et al. (2022) [11] | Analysis of Nigerian railway performance (1970-2010). | Data limited to historical analysis up to 2010. Nigeria only. |
| Аман (2023)[6] | Analysis of land transport (road and rail) in Ethiopia and multimodal transport networks. | Focus on Ethiopia without broader regional analysis. |
| African Infrastructure Database (AUDA-NEPAD)[7] | Tracks transport projects by country in Africa. | Lacks additional geographical and technical information. |
| African Regional Integration Index (ARII) [4b)] | Assesses status of transport and other infrastructure, aiming to use regional indicators. | Regional indicators, no geospatial high resolution data. No reliable data on regional indicators like cross-border connectivity. |
| African Infrastructure Knowledge Portal (AIKP) [4a)] | Provides data on ports and air transport capacity and freight value. | Limited access to data on ports and air transport by country. |
| MapAfrica [4c)] | Maps transport project nodes with risk categories, funders, and beneficiaries. | Misses vectorial details and edge measures of transport networks. |
| PIDA projects dashboard [8] | Visualizes PIDA projects by sector, country, and status on a map. | Limited to visualization; lacks detailed technical data. |
| TTTFP [25] | Maps major regional road corridors in Africa. | Lacks additional metadata and geopackage formats. |
| USGS geodatabase (Padilla et al., 2021) [21] | Comprehensive data on ports, roads, and railways in Africa. | Mineral-related only, may lack other transport information. |

| | | |
|---|---|---|
| World Bank report (2024) [30] | Assessment of corridors and monitoring institutions in Africa. | Specific corridors, no geographical database. |
| African Development Bank reports (2019, 2023) [1,2] | Details on cross-border road corridors, including project characteristics, length, and costs. | Focused on specific corridors; lacks broader regional integration data. |
| Thorn et al. (2022) [24] | Open-Source Geo-database of 184 projects, including railways, ports, airports, and industrial parks. | Focus on the 184 projects, no other transport infrastructures. |
| OurAirports (2025)[19] | Open-Source, global airports Geodatabase with size specification (very small, small, medium, etc.). | Does not contain information on the routes and capacity of airports. |

*Table S2 List of Sources for each dataset composing the database.*

| ***Dataset*** | ***Sources*** |
|---|---|
| Airport Network | World Bank Global Airports database [29] |
| | OurAirports [19] |
| Maritime Network | Verschuur, 2022 [28] |
| | USGS geodatabase (Padilla et al., 2021) [21] |
| | PortWatch[27] |
| | Thorn et al., 2022[24] |
| Inland Waterways (IWW) Network | OpenStreetMap Waterways for Africa[3] |
| | NBI Technical Reports[17] |
| | Pant et al. (2018) [22] |
| | Munyangeyo et al. (2022) [16] |
| | https://victoriatugandbarge.com/routes[14] |
| Rail Network | OSM[20] |
| | https://github.com/trg-rail/africa_rail_network[32] |
| | Thorn et al., 2022[24] |
| | AU-PIDA[8] |
| | CPCS, 2009[10] |
| Road Network | OSM[20] |
| | AfDB, 2023[2] |
| | AfDB, 2019[1] |
| | TTTFP[25] |

*Table S3 Values used to evaluate investment costs for rail lines.*

| ***Cost type*** | ***Cost unit*** | ***Cost mean*** | ***Cost min*** | ***Cost max*** | ***Source*** |
|---|---|---|---|---|---|
| Construction and upgrading | USD/km | 5,137,500 | 3,853,125 | 6,421,875 | Koks et al. 2019[13] |
| Capital and construction and upgrading | USD/km | 8,100,000 | 6,075,000 | 10,125,000 | Dulac et al. 2014[12] |
| Operations and Management | USD/km | 72,000 | 54,000 | 90,000 | Dulac et al. 2014[12] |

*Table S4 Comparison of length in km by country of our railway network dataset and the CIA[8] and WorldPop[30] Review figures. Percentage difference in length between the two sources with respect to our dataset is also reported.*

| Country | Railway network database | CIA | WorldPop Review | db vs CIA | db vs WorldPop |
|---|---|---|---|---|---|
| Eritrea | 122 | 306 | 306 | -151% | -151% |
| Ghana | 421 | 947 | 947 | -125% | -125% |
| Sudan | 4,235 | 7,251 | 7,251 | -71% | -71% |
| Mozambique | 2,893 | 4,787 | 4,787 | -65% | -65% |
| Kenya | 2,720 | 3,819 | 3,819 | -40% | -40% |
| Liberia | 324 | 429 | 429 | -33% | -33% |
| South Africa | 25,040 | 30,400 | 20,986 | -21% | 16% |
| Zambia | 2,725 | 3,126 | 3,126 | -15% | -15% |
| Senegal | 838 | 906 | 906 | -8% | -8% |
| South Sudan | 235 | 248 | 248 | -6% | -6% |
| Zimbabwe | 3,241 | 3,427 | 3,427 | -6% | -6% |
| Djibouti | 93 | 97 | 97 | -4% | -4% |
| Egypt | 4,986 | 5,085 | 7,024 | -2% | -41% |
| Côte d'Ivoire | 648 | 660 | 660 | -2% | -2% |
| Angola | 2,738 | 2,761 | 2,761 | -1% | -1% |
| Gabon | 654 | 649 | 649 | 1% | 1% |
| Mali | 600 | 593 | 593 | 1% | 1% |
| Uganda | 1,261 | 1,244 | 1,244 | 1% | 1% |
| Burkina Faso | 634 | 622 | 622 | 2% | 2% |
| Cameroon | 1,018 | 987 | 977 | 3% | 4% |
| Democratic Republic of the Congo | 4,242 | 4,007 | 4,007 | 6% | 6% |
| Mauritania | 781 | 728 | 728 | 7% | 7% |
| Benin | 480 | 438 | 438 | 9% | 9% |
| Nigeria | 4,216 | 3,798 | 3,798 | 10% | 10% |
| Namibia | 2,955 | 2,628 | 2,628 | 11% | 11% |
| Eswatini | 342 | 301 | 301 | 12% | 12% |
| Botswana | 1,014 | 888 | 888 | 12% | 12% |
| Tunisia | 2,487 | 2,173 | 2,173 | 13% | 13% |
| Algeria | 4,623 | 4,020 | 4,560 | 13% | 1% |
| DRC | 592 | 510 | 510 | 14% | 14% |
| Malawi | 990 | 767 | 767 | 23% | 23% |
| Togo | 739 | 568 | 568 | 23% | 23% |
| Morocco except Western Sahara | 2,776 | 2,067 | 2,109 | 26% | 24% |
| Ethiopia | 980 | 659 | 659 | 33% | 33% |
| Guinea | 1,717 | 1,086 | 1,086 | 37% | 37% |
| Tanzania | 6,637 | 4,097 | 4,097 | 38% | 38% |
| Lesotho | 2 | | 2 | | -25% |
| Niger | 148 | | | | |
| Sierra Leone | 198 | | 84 | | 57% |
| Total | 99,365 | 97,079 | 59,882 | 2% | 40% |

*Table S5 Comparison of length in km by country of our road network dataset and the HeiGIT dataset (Randhawa et al., 2025)[23]. Percentage difference in length between the source with respect to our dataset is also reported.*

| Country | HeiGIT lengths (km) | | Our database lengths (km) | | Length Difference (%) | |
|---|---|---|---|---|---|---|
| | Paved | Unpaved | Paved | Unpaved | Paved | Unpaved |
| Algeria | 47,173 | 2,135 | 46,960 | 2,348 | 0.45 | -9.08 |
| Angola | 11,468 | 8,341 | 11,595 | 8,214 | -1.09 | 1.54 |
| Benin | 2,589 | 2,841 | 3,168 | 2,262 | -18.27 | 25.58 |
| Botswana | 7,370 | 1,969 | 7,367 | 1,972 | 0.04 | -0.17 |
| Burkina Faso | 3,932 | 4,673 | 4,015 | 4,590 | -2.07 | 1.81 |
| Burundi | 1,442 | 3,537 | 1,439 | 3,540 | 0.22 | -0.09 |
| Cabo Verde | 542 | 32 | 414 | 159 | 30.91 | -80.23 |
| Cameroon | 6,477 | 10,712 | 6,724 | 10,465 | -3.68 | 2.36 |
| Central African Republic | 785 | 9,972 | 893 | 9,864 | -12.08 | 1.09 |
| Chad | 2,187 | 8,385 | 2,276 | 8,297 | -3.88 | 1.07 |
| Comoros | 89 | 32 | 101 | 19 | -11.86 | 61.69 |
| Congo | 1,686 | 1,527 | 1,705 | 1,508 | -1.10 | 1.25 |
| Côte d'Ivoire | 6,233 | 4,815 | 6,775 | 4,272 | -8.01 | 12.70 |
| Democratic Republic of the Congo | 3,231 | 29,329 | 4,380 | 28,179 | -26.25 | 4.08 |
| Djibouti | 483 | 247 | 474 | 255 | 1.84 | -3.41 |
| Egypt | 21,137 | 727 | 20,653 | 1,211 | 2.34 | -39.96 |
| Equatorial Guinea | 1,767 | 49 | 1,767 | 49 | 0.00 | 0.00 |
| Eritrea | 414 | 870 | 419 | 864 | -1.31 | 0.63 |
| Ethiopia | 10,246 | 8,558 | 10,484 | 8,320 | -2.27 | 2.86 |
| Gabon | 2,386 | 2,962 | 2,388 | 2,959 | -0.10 | 0.08 |
| Gambia | 851 | 223 | 923 | 150 | -7.89 | 48.48 |
| Ghana | 6,402 | 2,139 | 6,427 | 2,113 | -0.40 | 1.21 |
| Guinea | 2,493 | 4,644 | 2,618 | 4,519 | -4.78 | 2.77 |
| Guinea-Bissau | 581 | 239 | 585 | 234 | -0.71 | 1.77 |
| Kenya | 7,529 | 8,917 | 8,284 | 8,162 | -9.11 | 9.25 |
| Lesotho | 1,498 | 676 | 1,643 | 532 | -8.80 | 27.18 |
| Liberia | 758 | 2,104 | 768 | 2,094 | -1.33 | 0.49 |
| Libya | 18,036 | 2,322 | 18,118 | 2,241 | -0.45 | 3.65 |
| Madagascar | 2,766 | 2,800 | 2,753 | 2,813 | 0.49 | -0.48 |
| Malawi | 2,259 | 1,792 | 2,261 | 1,790 | -0.09 | 0.11 |
| Mali | 4,992 | 7,887 | 5,076 | 7,803 | -1.65 | 1.08 |
| Mauritania | 4,671 | 740 | 4,673 | 737 | -0.05 | 0.33 |
| Mauritius | 1,040 | 0 | 1,040 | 0 | 0.00 | 0.00 |
| Morocco | 33,658 | 1,073 | 33,921 | 810 | -0.78 | 32.57 |
| Mozambique | 7,656 | 8,102 | 7,623 | 8,135 | 0.44 | -0.41 |
| Namibia | 8,948 | 30,957 | 9,053 | 30,851 | -1.17 | 0.34 |
| Niger | 4,132 | 4,501 | 4,265 | 4,367 | -3.13 | 3.06 |
| Nigeria | 37,341 | 11,031 | 37,981 | 10,390 | -1.69 | 6.17 |
| Rwanda | 1,596 | 3,901 | 1,641 | 3,856 | -2.72 | 1.16 |
| Sao Tome and Principe | 146 | 17 | 143 | 20 | 1.97 | -14.08 |
| Senegal | 6,050 | 4,478 | 6,201 | 4,327 | -2.43 | 3.49 |
| Seychelles | 46 | 0 | 46 | 0 | 0.00 | 0.00 |
| Sierra Leone | 1,323 | 2,013 | 1,287 | 2,049 | 2.79 | -1.75 |
| Somalia | 1,484 | 6,953 | 1,555 | 6,882 | -4.52 | 1.02 |
| South Africa | 73,411 | 26,307 | 74,375 | 25,344 | -1.30 | 3.80 |

| | | | | | | |
|---|---|---|---|---|---|---|
| South Sudan | 236 | 4,328 | 264 | 4,300 | -10.83 | 0.67 |
| Sudan | 7,803 | 6,378 | 7,773 | 6,408 | 0.39 | -0.47 |
| Swaziland | 1,499 | 618 | 1,499 | 619 | 0.04 | -0.09 |
| Togo | 1,533 | 2,151 | 1,539 | 2,145 | -0.42 | 0.30 |
| Tunisia | 8,291 | 795 | 7,299 | 1,787 | 13.59 | -55.50 |
| Uganda | 4,562 | 7,085 | 4,480 | 7,167 | 1.83 | -1.15 |
| United Republic of Tanzania | 10,600 | 21,917 | 11,110 | 21,406 | -4.60 | 2.39 |
| Zambia | 8,084 | 3,900 | 8,013 | 3,971 | 0.89 | -1.80 |
| Zimbabwe | 9,028 | 1,979 | 9,013 | 1,994 | 0.17 | -0.75 |
| **Total** | **412,938** | **284,678** | **418,248** | **279,369** | **-0.01** | **0.02** |

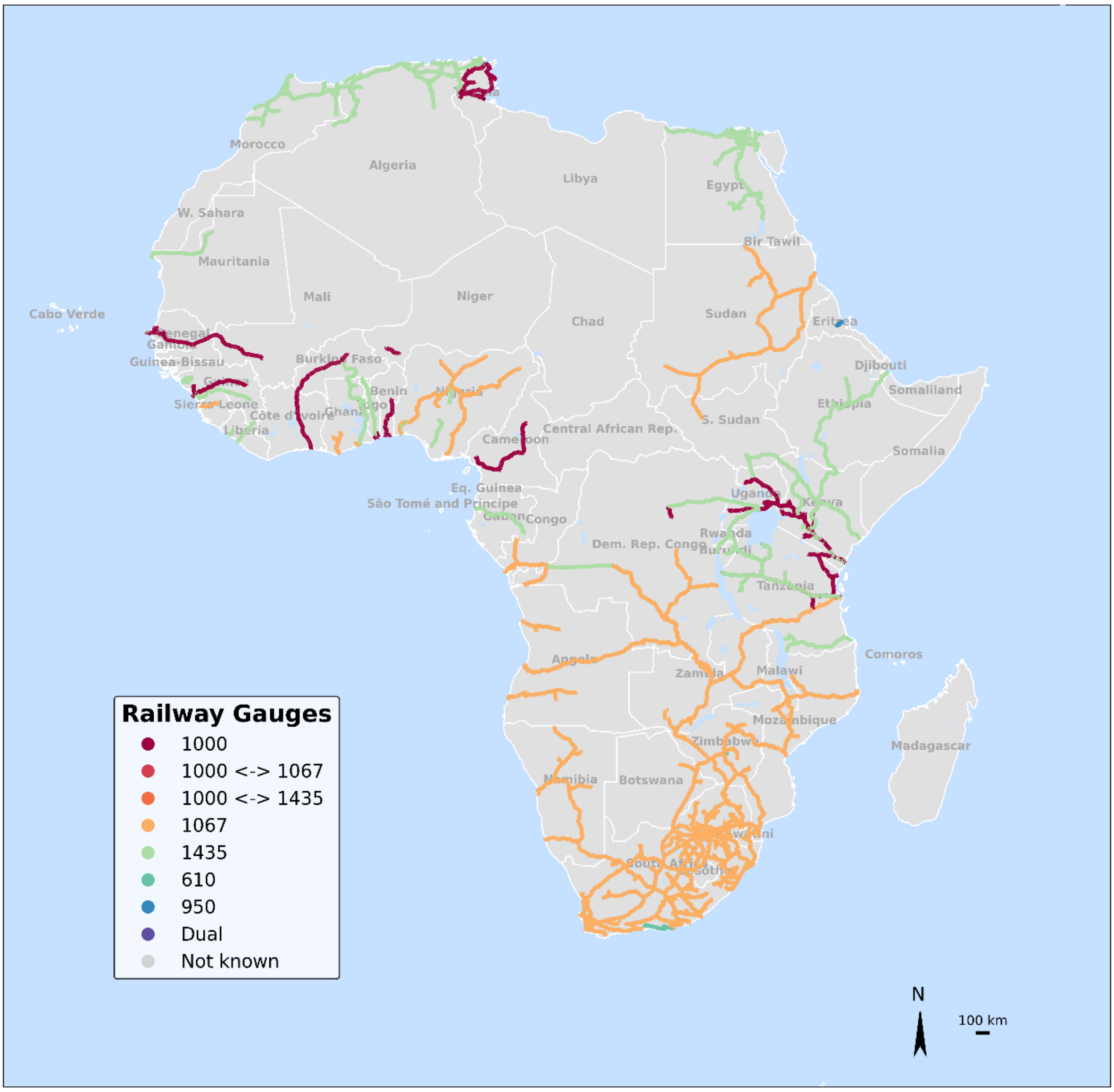


*Figure S1 – Railways network distinguished by line name by Open Street Map.*

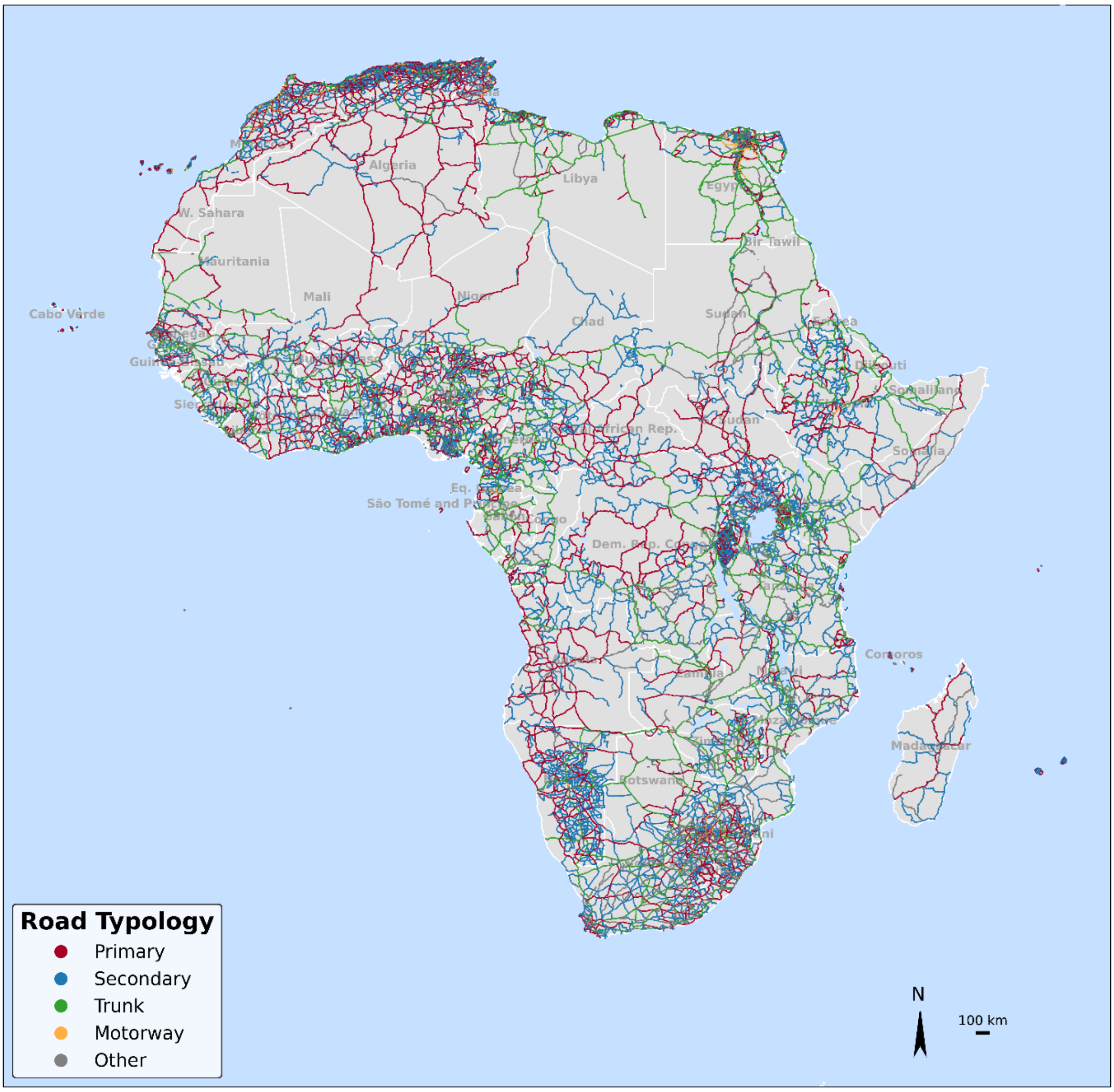


*Figure S2 – Road network classification by OpenStreetMap "highway" tag.*

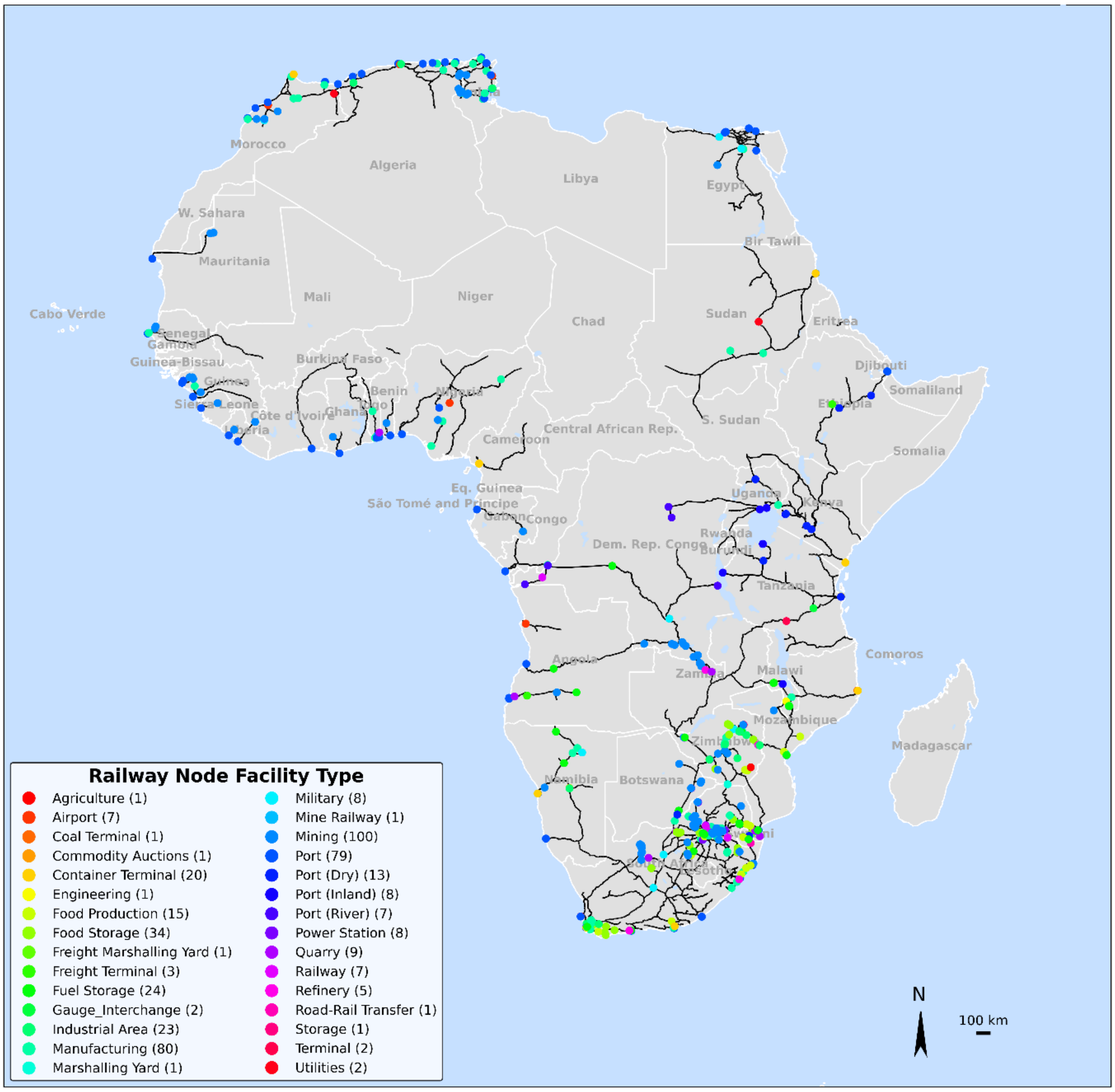


*Figure S3 - Map representation of important railway station showing their "facility" tag types and numbers. These facilities represent locations where the rail network to connected to locations of important socio-economic activities.*

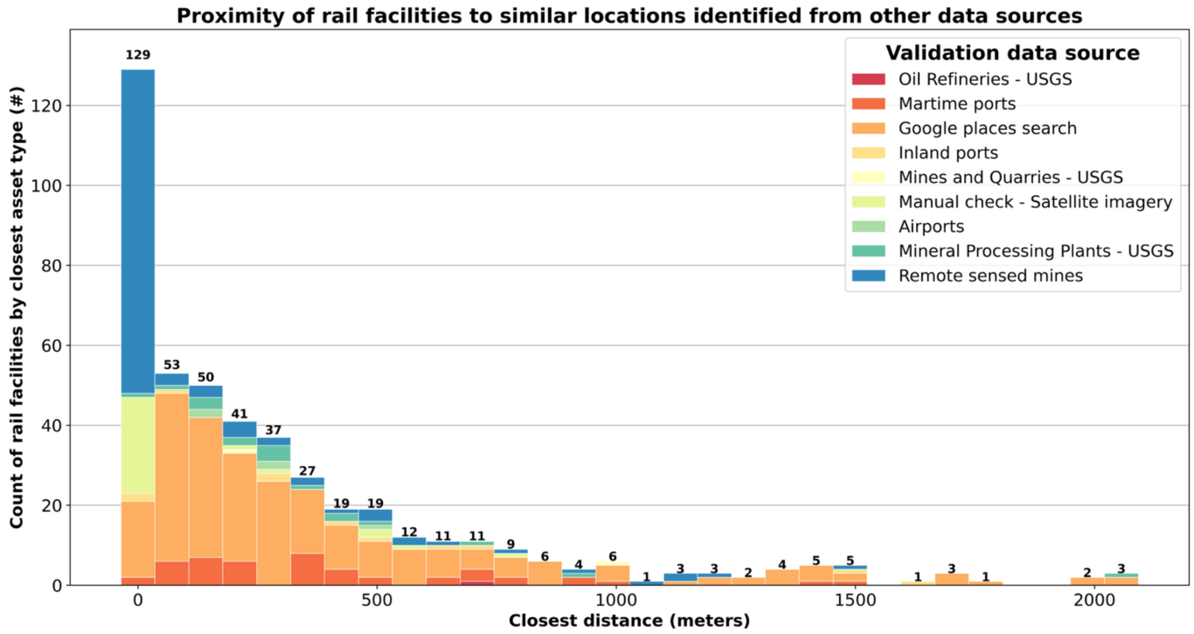


*Figure S4 - Histogram showing the proximity of the rail facilities to similar locations identified from multiple data sources for validation.*

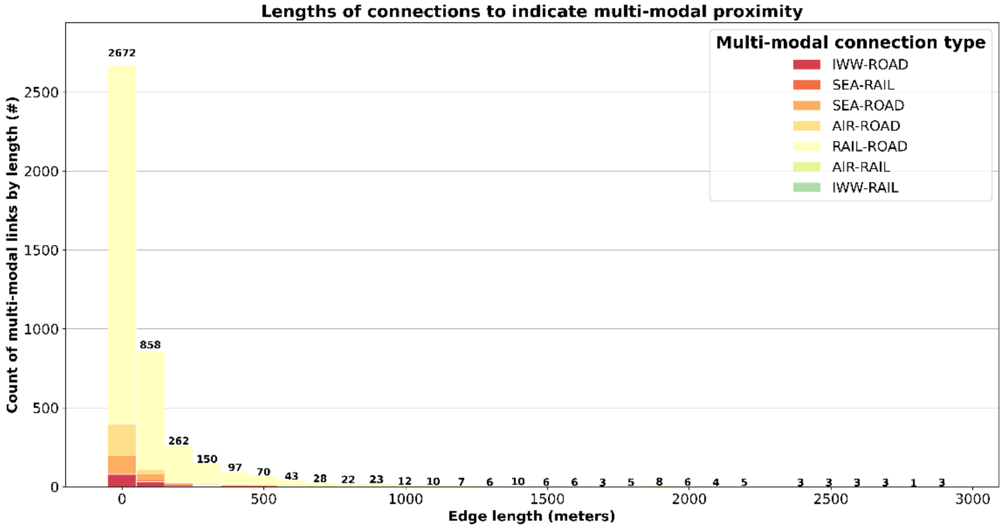


*Figure S5 - Histogram the lengths of multimodal connections between different type of transport modes. These lengths are representative of the spatial proximity of two intermodal connections.*

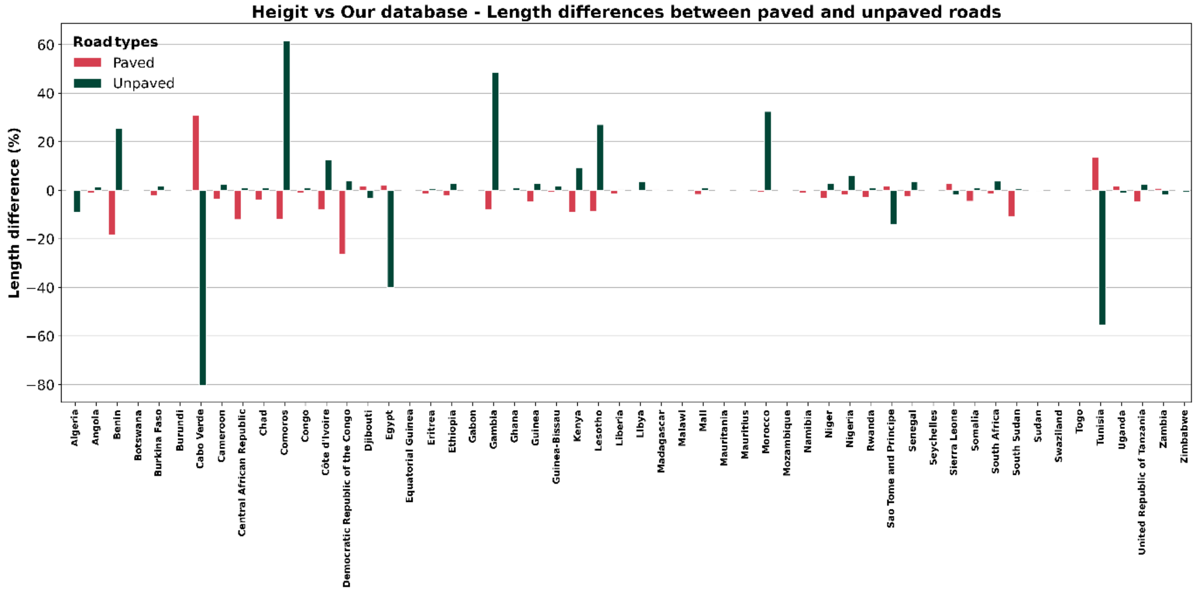


*Figure S6 - Bar plots showing the difference between the HeiGIT road lengths and our database road lengths as a percentage of our road lengths for paved and unpaved roads across Africa. Negative values here show that we are overpredicting the values compared to the HeiGIT data, and positive values show that the HeiGIT data overpredicts values compared to our database.*

## References


1. AfDB. "Cross-border Road Corridors: The Quest to Integrate Africa." (2019).
2. AfDB. “Expanding Market Access in Africa and Nurturing Continental Integration.” (2023).
3. Africa Geoportal. OpenStreetMap Waterways for Africa. https://africageoportal.maps.arcgis.com/home/item.html?id=82232d0415c04e7086414dff7eb1310f (accessed August 2025).
4. African Union, AfDB, UNECA. (2019). Africa regional integration index report 2019. https://www.integrate-africa.org/fileadmin/uploads/afdb/Documents/ARII-Report2019-FIN-R40-11jun20.pdf
5. African Development Bank. (n.d.)
   a) AIKP. Infrastructure Africa. Retrieved February 2025, from https://infrastructureafrica.opendataforafrica.org/
   b) ARII. Integrate Africa. Retrieved February 2025, from https://www.integrate-africa.org/
   c) MapAfrica. Retrieved February 2025, from https://mapafrica.afdb.org/en
6. Amah, A.M., 2023. Challenges and Prospects of Multimodal Transport System in Ethiopia. BRICS Transport, 2(2): 1-6.
7. AUDA-NEPAD. African Union Development Agency. (n.d.). African Infrastructure Map. African Infrastructure Database. Retrieved November 2024, from https://aid.nepad.org/welcome/project-mapping/ African Regional Integration Index (ARII)
8. AU-PIDA. African Union. (n.d.). PIDA projects. AU-PIDA. https://www.au-pida.org/pida-projects/
9. Central Intelligence Agency (CIA). The World Factbook (online edition). Washington, DC: CIA, 2025. https://www.cia.gov/the-world-factbook/ (accessed June 2025).
10. CPCS Transcom International Limited. (2009). East African Railways Master Plan Study: Final Report. Prepared for the East African Community.
11. Ogochukwu, C. G., Ogochukwu, O. F., Ogorchukwu, I. M., & Ebuka, I. A. Assessment of the performance of railway transportation in Nigeria from 1970 to 2010. Scientific African, 15, e01120. https://doi.org/10.1016/j.sciaf.2022.e01120. (2022).
12. Dulac, John. "Global land transport infrastructure requirements." Paris: International Energy Agency 20 (2013): 2014.
13. Koks EE, Rozenberg J, Zorn C, Tariverdi M, Vousdoukas M, Fraser SA, et al. A global multi-hazard risk analysis of road and railway infrastructure assets. Nature Communications. 2019; 10(1): p. 2677.
14. Lake Victoria Routes. https://victoriatugandbarge.com/routes (accessed August 2025).
15. Mphigalale, T.V. Infrastructure investment in Sub-Saharan Africa: Opportunities, risks and prospects for economic development (2020).

16. Munyangeyo, A., & Gudmestad, O. T. Safety aspects for inland personnel transport; case study Congo River. In ISOPE International Ocean and Polar Engineering Conference (pp. ISOPE-I). ISOPE (2022).
17. NBI Technical Reports - WRM-2022-02. Nile River Navigation - Integration of scenarios for sector development into the Strategic Water Resources Analysis. https://nilebasin.org/sites/default/files/2023-09/WRM-2022-02_Nile%2520River%2520Navigation.pdf (2022).
18. Obeng, D.A., Bessah, E., Amponsah, W., Dzisi, E.K. and Agyare, W.A. Ghana's railway transport services delivery: A review. Transportation Engineering, 8: 100111 (2022).
19. OurAirports. Airport data. https://ourairports.com/ (accessed June 2025).
20. Open Street Maps. Open Street Maps. https://download.geofabrik.de/ (2021).
21. Padilla, A. D., Otarod, D., Deloach-Overton, S. W., Kemna, R. F., Freeman, P. A., Wolfe, E. R., ... & Brioche, A. S. Compilation of geospatial data (GIS) for the mineral industries and related infrastructure of Africa. US Geological Survey. https://doi.org/10.5066/P97EQWXP (2021).
22. Pant, R., Koks, E. E., Russell, T., & Hall, J. W. Transport Risks Analysis for The United Republic of Tanzania – Systemic vulnerability assessment of multi-modal transport networks. Final Report Draft. Oxford, UK (2018).
23. Randhawa, S., Aygün, E., Randhawa, G., Herfort, B., Lautenbach, S., & Zipf, A. (2025). Paved or unpaved? A deep learning derived road surface global dataset from mapillary street-view imagery. ISPRS Journal of Photogrammetry and Remote Sensing, 223, 362-374.
24. Thorn, J. P.R., Mwangi, B.; Juffe Bignoli, D., *The African Development Corridors Database.* Dryad, Dataset. https://doi.org/10.5061/dryad.9kd51c5hw (2022).
25. Tripartite Transport and Transit Facilitation Programme (TTTFP). https://tttfp.org/corridors/all-corridors/ (2019).
26. UNCTAD. United Nations Conference on Trade and Development. (n.d.). Trade-and-Transport Dataset (experimental). UNCTADstat. Retrieved Nov 2024, from https://unctadstat.unctad.org/EN/Index.html
27. UN Global Platform; IMF *PortWatch* https://portwatch.imf.org/
28. Verschuur, J. Global multi-hazard risk to port infrastructure and trade. Mendeley Data. V1, doi: 10.17632/kdyt24tsh5.1 (2022).
29. World Bank Group. Global Airports: Locations of airports with international travel. https://datacatalog.worldbank.org/search/dataset/0038117/Global-Airports (2020).
30. World Bank. Toward a Data-driven Understanding of Trade and Transport Corridors - An Assessment of the Potential of the Existing Transport Corridor Monitoring Systems to Foster Policy Dialogue and to Strengthen Corridor Management Institutions in Africa (English). Sub-Saharan Africa Transport Policy Program (SSATP) working paper series Washington, D.C. : World Bank Group. http://documents.worldbank.org/curated/en/099062624065590055/P1747631502efe01918fc51d71b34b63f01. (2024) African Development Bank reports (2019, 2023).

31. World Population Review. World Population Review. https://worldpopulationreview.com/ (accessed June 2025).
32. Young, M. *An Open Source Routable Rail Dataset For Africa*. https://github.com/trg-rail/africa_rail_network
